# Naringenin confers defence against *Phytophthora nicotianae* through antimicrobial activity and induction of pathogen resistance in tobacco

**DOI:** 10.1111/mpp.13255

**Published:** 2022-09-12

**Authors:** Mingming Sun, Lei Li, Chengdong Wang, Luanming Wang, Di Lu, Danyu Shen, Jie Wang, Caihong Jiang, Lirui Cheng, Xuhao Pan, Aiguo Yang, Yuanying Wang, Xiaowei Zhu, Bin Li, Yiting Li, Feng Zhang

**Affiliations:** ^1^ Key Laboratory of Tobacco Genetic Improvement and Biotechnology, Tobacco Research Institute Chinese Academy of Agricultural Sciences Qingdao China; ^2^ China Tobacco Shandong Industrial Co., Ltd. Jinan China; ^3^ College of Plant Protection Nanjing Agricultural University Nanjing China; ^4^ Chongqing Tobacco Corporation Chongqing China; ^5^ Sichuan Tobacco Corporation Chengdu China; ^6^ The Key Laboratory of Plant Immunity Nanjing Agricultural University Nanjing China

**Keywords:** antimicrobial activity, naringenin, *NtCHS*, oomycete, *Phytophthora nicotianae*, plant defence

## Abstract

Tobacco black shank caused by *Phytophthora nicotianae* is a serious disease in tobacco cultivation. We found that naringenin is a key factor that causes different sensitivity to *P*. *nicotianae* between resistant and susceptible tobacco. The level of basal flavonoids in resistant tobacco was distinct from that in susceptible tobacco. Of all flavonoids with different content, naringenin showed the best antimicrobial activity against mycelial growth and sporangia production of *P*. *nicotianae* in vitro. However, naringenin showed very low or no antimicrobial activity to other plant pathogens. We found that naringenin induced not only the accumulation of reactive oxygen species, but also the expression of salicylic acid biosynthesis‐related genes. Naringenin induced the expression of the basal pathogen resistance gene *PR1* and the *SAR8.2* gene that contributes to plant resistance to *P. nicotianae*. We then interfered with the expression of the chalcone synthase (*NtCHS*) gene, the key gene of the naringenin synthesis pathway, to inhibit naringenin biosynthesis. *NtCHS*‐RNAi rendered tobacco highly sensitive to *P*. *nicotianae*, but there was no change in susceptibility to another plant pathogen, *Ralstonia solanacearum*. Finally, exogenous application of naringenin on susceptible tobacco enhanced resistance to *P*. *nicotianae* and naringenin was very stable in this environment. Our findings revealed that naringenin plays a core role in the defence against *P*. *nicotianae* and expanded the possibilities for the application of plant secondary metabolites in the control of *P. nicotianae*.

## Graphical abstract

This study demonstrates that the plant secondary metabolite naringenin specifically inhibits *Phytophthora nicotianae*.

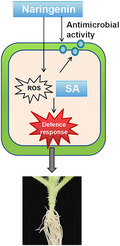

## 1 INTRODUCTION

Plant‐pathogenic oomycetes pose a serious threat to agriculture, horticulture, forestry, aquaculture and natural ecosystems (Wang, Tyler, et al., 2019). They encompass over 100 species that cause devastating diseases in both plants and animals. The *Phytophthora* species are the most serious pathogens. They infect a broad group of hosts and cause diseases such as root rot, late blight, downy blight, and downy mildew (Derevnina et al., 2016; Kamoun et al., 2015; Panabieres et al., 2016). Tobacco black shank (TBS) disease, caused by *Phytophthora nicotianae,* threatens production in tobacco‐producing areas across the world (Gallup et al., 2018). Infections mainly occur in the adult stage of tobacco, resulting in significant yield loss and quality reduction. Roots and stems are the main parts infected, leading to progressive decay of the diseased tissues, such as root and stem necrosis, wilting, and chlorosis, and finally death (Chen et al., 2020; Csinos & Minton, 1983; Gallup et al., 2006). Mycelia, oospores and chlamydospores from *P*. *nicotianae* are found in soil and plant tissues in compost through the winter and can survive for more than 3 years. Soil is therefore the main source of infection, while manure and irrigation promote the spread of pathogens (Antonopoulos et al., 2010; Gallup et al., 2018).

TBS is a very difficult disease to control due to the diversity and high adaptability of *P. nicotianae* (Gallup et al., 2018; Ji et al., 2014). Its management relies on the integration of different approaches, including planting resistant varieties, fungicide applications, crop rotation, management of water and fertilizer (Shew & Lucas, 1991). So far, four physiological races (0, 1, 2 and 3) of *P*. *nicotianae* have been reported. The undomesticated *Nicotiana* species are an important source of resistant varieties. Tobacco cultivars resistant to race 0 of *P*. *nicotianae* conferred by the genes *Php* or *Phl* have been widely used across the world; however, cultivars have become susceptible to *P*. *nicotianae* as there has been a gradual shift of pathogen populations from race 0 to race 1, making the strategy of TBS‐resistant cultivars ineffective in disease control (Johnson et al., 2002; Li et al., 2006; Sullivan et al., 2005). In addition, *P*. *nicotianae* has a large arsenal of secreted proteins, termed effectors, that act as weapons to overcome the host resistance and promote infection (Hou et al., 2019; Lee et al., 2018). These are the main reasons that crop rotation is highly recommended to avoid disease outbreaks and propagation of *P*. *nicotianae* in the field (Yong et al., 2010). Pesticide application is another important approach to managing TBS. Several fungicides such as mefenoxam and potassium phosphite have been applied for many years but resistance in *Phytophthora* has been reported for both compounds (Hao et al., 2019; Ji et al., 2014). Some new fungicides against oomycetes, such as fluopicolide, ethaboxam, oxathiapiprolin and mandipropamid, with different modes of action have been developed and they have shown higher efficiency in treatment of *Phytophthora* (Hao et al., 2019; Ji et al., 2014; Qu et al., 2016). These new fungicides provide another highly effective approach against pathogens in addition to rotation and proper use of all existing fungicides. However, more attention should be paid to the development of resistance to these new fungicides among the populations of *P. nicotianae* (Panabieres et al., 2016; Qu et al., 2016).

Biocontrol has been shown to be an alternative way to manage TBS efficiently, mainly using biocontrol strains and new compounds that have been isolated from natural organisms. Among biocontrol strains, *Pseudomonas fluorescens* and *Bacillus* spp. have been widely studied and used due to the antimicrobial components they release, such as peptides and enzymes (Choudhary & Johri, 2009; Fira et al., 2018; Rajaofera et al., 2018). These antimicrobial components can improve plant growth, as well as induce a plant immune response to abiotic stress (Choudhary & Johri, 2009; Sun et al., 2017). In addition, some biocontrol strains, such as the plant growth‐promoting rhizobacterium *Bacillus amyloliquefaciens* Ba168, secrete proteins and peptides that target pathogens directly, for example, by damaging the cell wall and membrane of *P*. *nicotianae* (Guo et al., 2020). In addition, various natural agents produced from plants are another source of antimicrobial components. Some secondary metabolites are not involved in growth, development or reproduction of plants directly. Rather, they perform special functions under a given set of conditions such as pathogen attack, water deficit or extreme temperature (Bartwal et al., 2012). The essential oil eugenol and diallyl disulphides have been confirmed to be effective against TBS by destroying mycelial cell membrane integrity, causing an increase in cell membrane permeability and leading to cell death (Jing et al., 2017; Wang et al., 2019). Phytoalexins with antiseptic, anti‐inflammatory, antioxidant or antimicrobial activities accumulate soon after pathogen infection (Chripkova et al., 2016; Kumar et al., 2006).

Over 9000 categories of flavonoids have been found in various plants, making them one of the largest families of secondary metabolites (Wang et al., 2011). They are essential factors that are involved in aspects of plant development and defence, and flower and fruit quality (Treutter, 2005; Wang et al., 2011). Flavonoids often accumulate in specialized cells and there are only a few studies on their function against pathogens (Kariu et al., 2017; Martínez‐Castillo et al., 2018; Paczkowski et al., 2017; Tattini et al., 2004). Naringenin is one of the major flavonoids and is mainly found in citrus fruits, including tangerine, lemon, orange and grapefruit (Manchope et al., 2017). Naringenin accumulates when plants are infected by *Rhizobium leguminosarum* bv. *viceae*, *Pseudomonas syringae* pv. *pisi* or *Plasmodiophora brassicae* (Makarova et al., 2016; Paesold et al., 2010). In addition, naringenin has shown anti‐inflammatory, antiviral and antifungal activities, for example against *Fusarium* spp. including *F*. *poae*, *F*. *culmorum* and *F*. *graminearum*, and the rice pathogen *Magnaporthe grisea* (Den Hartogh & Tsiani, 2019; Padmavati et al., 1997; Skadhauge et al., 1997). However, knowledge of the mechanism of how naringenin contributes to defence against *P*. *nicotianae* is still unknown. We found that naringenin conferred defence against *P*. *nicotianae* by inhibition of mycelial growth and sporangia production. Through metabolomics analysis, we found that basal flavanones and flavonols in resistant tobacco varieties were distinct from those in susceptible tobacco varieties. Of all flavanones and flavonols, we discovered that naringenin inhibited *P*. *nicotianae* most effectively. However, naringenin showed no or very low inhibition activity towards other plant pathogens. We also found that naringenin induced plant resistance to *P*. *nicotianae* by inducing the accumulation of O_2_^−^ and H_2_O_2_, the expression of genes regulating salicylic acid (SA) biosynthesis and SA signalling, and the expression of genes regulating plant basal defence and resistance to *P*. *nicotianae*. Knockdown of chalcone synthase (*CHS*), a gene encoding a rate‐limiting enzyme of the naringenin sythesis pathway, rendered tobacco highly susceptible to *P*. *nicotianae*, but it kept the same susceptibility to other plant pathogens. Interestingly, exogenous application of naringenin to tobacco enhanced resistance to TBS without affecting the agronomic traits. Our findings reveal that naringenin showed antimicrobial activity towards *P*. *nicotianae* and is therefore a potential botanical pesticide in tobacco disease control for the future.

## 2 RESULTS

### 2.1 Metabolite profiling of basal flavanone and flavonol in resistant tobacco varieties is distinct from that in susceptible tobacco varieties

**FIGURE 1:**
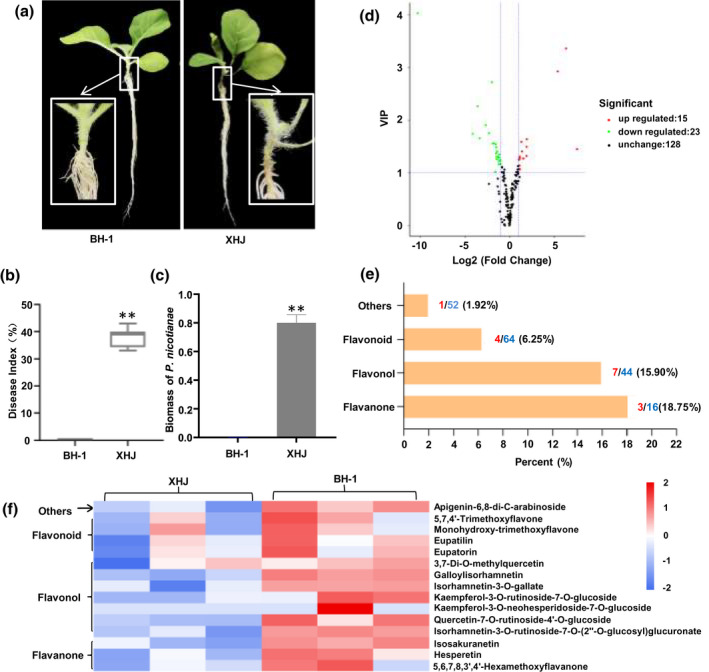
Metabolite profiling of resistant (BH‐1) and susceptible (XHJ) tobacco lines in response to *Phytophthora nicotianae*. Disease symptoms observed following root inoculation with a spore suspension of *P. nicotianae* at 5 days postinoculation (dpi). (a) Phenotypes of BH‐1 and XHJ in response to *P. nicotianae*. (b) Disease index of *P. nicotianae* on BH‐1 and XHJ (*n* = 3, error bars, *SD*). XHJ is significantly different from BH‐1 according to a Mann–Whitney test (**p < 0.01). (c) Biomass of *P. nicotianae* in BH‐1 and XHJ (*n* = 3, error bars, *SD*). XHJ is significantly different  from BH‐1 by a Mann–Whitney test (**p < 0.01). (d) A volcano plot of metabolites of BH‐1 compared to XHJ. (e) The proportion of the number with a higher content of metabolites in each class of metabolites. The red numbers indicate the number of metabolites with significantly higher content, and the blue numbers indicate the total number of the metabolites detected in the metabolome. (f) A heatmap of 15 metabolites with higher content in BH‐1 than XHJ. The left three columns are from roots of XHJ and the right three columns are from roots of BH‐1; each column represents a biological replicate.

Typical resistant and susceptible cultivated tobacco varieties to TBS were used in this study. The typical cultivated tobacco varieties in China are Beinhart 1000‐1 (BH‐1) and Xiaohuangjin 1025 (XHJ), which show resistance and susceptibility to *P*. *nicotianae*, respectively (Figure 1a). The disease index of BH‐1 is significantly lower than that of XHJ after inoculation with *P*. *nicotianae* (Figure 1b), and the colonization of *P*. *nicotianae* in XHJ is higher than that in BH‐1 (Figure 1c). To test whether there is a difference in the basal level of metabolites between the resistant and susceptible tobacco varieties in uninfected roots, the flavonoids of BH‐1 and XHJ were analysed by liquid chromatography‐electrospray ionization‐tandem mass spectrometry (LC‐ESI‐MS/MS) to evaluate the metabolite profiles. As shown in Table S1, 166 metabolites were identified and characterized by its distinct retention time and mass‐to‐charge ratio (*m*/*z*). Compared to XHJ, the content of 15 metabolites in BH‐1 were significant lower when considering fold change and the variable importance in projection (VIP) value of an orthogonal partial least‐squares discriminant analysis (OPLS‐DA) model (fold change > 2, VIP > 1) (Figure 1d and Table S2). These metabolites could be divided into four categories: flavonoids, flavonols, flavanones and others; the proportion of metabolites with a higher content of flavonoids was different in each type of metabolite (Figure 1e,f). The proportions of flavanones and flavonols were 18.75% and 15.90%, respectively. Through metabolite analysis, 16 flavanones were characterized. The content of nine flavanones, including liquiritigenin and naringenin, was slightly higher and the content of three flavanones, including isosakuranetin and hesperetin, was significantly higher in BH‐1 compared to XHJ (Table S3). We also characterized 44 flavonols, of which  the content of seven was significantly higher in BH‐1 than XHJ, such as kaempferol and quercetin (Table S4). These results indicate that there is a significant difference in the basal level of flavonoids between resistant and susceptible tobacco varieties, and this difference might be related to resistance to *P*. *nicotianae* in the plants.

### 2.2 Naringenin showed antimicrobial activity on *P. nicotianae* in vitro

**FIGURE 2:**
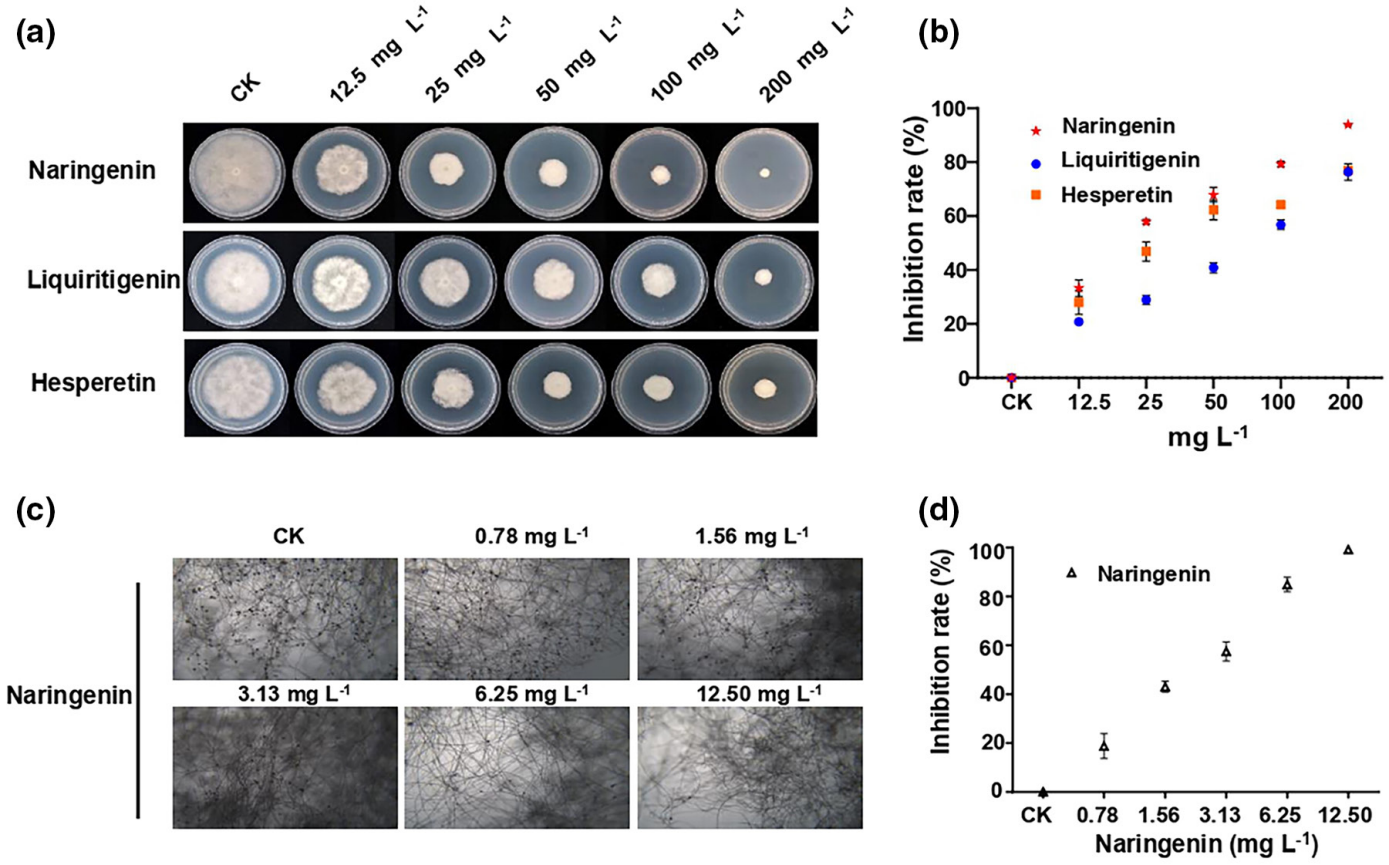
The antimicrobial activity of the different flavonoids on *Phytophthora nicotianae*. The control (CK) was added ethanol only. (a) Colony morphology of *P. nicotianae* on potato dextrose agarose amended with naringenin, liquiritigenin or hesperetin at 28°C for 7 days. (b) The inhibition rate of naringenin, liquiritigenin and hesperetin on mycelial growth (*n* = 3, error bars = *SD*). (c) The sporangia production of *P. nicotianae* is dramatically inhibited by naringenin under microscopic observation. (d) The inhibition rate of naringenin on the sporangia production (*n* = 3, error bars = *SD*).

For the 16 flavanones and 44 flavonols described above, we tested their antimicrobial activity against *P*. *nicotianae* in vitro. *P*. *nicotianae*  grew on potato dextrose agar (PDA) equally well in the presence of different concentrations of flavonoids. Only three flavanones inhibited the growth of *P*. *nicotianae*, and of these naringenin showed the strongest antimicrobial activity (Figure 2a,b). The half‐maximum effective concentration (EC_50_) of naringenin for inhibition of *P*. *nicotianae* mycelial growth was 22.01 mg/L, whereas the EC_50_ values of liquiritigenin and hesperetin were 51.43 and 30.20 mg/L (Table S5). In contrast, none of the flavonols, such as kaempferol, quercetin or reutinum, showed any inhibitory activity on the mycelial growth of even at concentrations up to 200 mg/L (Figure S1a,b and Table S5). Naringenin also inhibited growth of other oomycetes, such as *Phytophthora capsici*, with an EC_50_ value of 50.11 mg/L, but showed low inhibition activity on *Pythium aphanidermatum* (Figure S2 and Table S6). However, naringenin showed very low or no inhibitory activity on other plant pathogens such as *Sclerotinia sclerotiorum*, *Botrytis cinerea*, and *Fusarium graminearum* (Figure S3). We next tested whether naringenin affected the reproduction of *P*. *nicotianae* by microscopic observation. As shown in Figure 2c,d and Table S6, the EC_50_ and EC_90_ values of naringenin for inhibition of *P*. *nicotianae* sporangia production were 2.01 mg/L and 6.62 mg/L, respectively. These values are significantly lower than the EC_50_ for mycelial growth. Furthermore, we used reverse transcription‐quantitative PCR (RT‐qPCR) to confirm that cell growth and reproduction‐related genes were down‐regulated when *P*. *nicotianae* was treated with naringenin (Figure S4). These results indicate that naringenin inhibits not only the mycelial growth but also the reproduction of *P*. *nicotianae*.

### 2.3 Naringenin induces accumulation of reactive oxygen species

**FIGURE 3:**
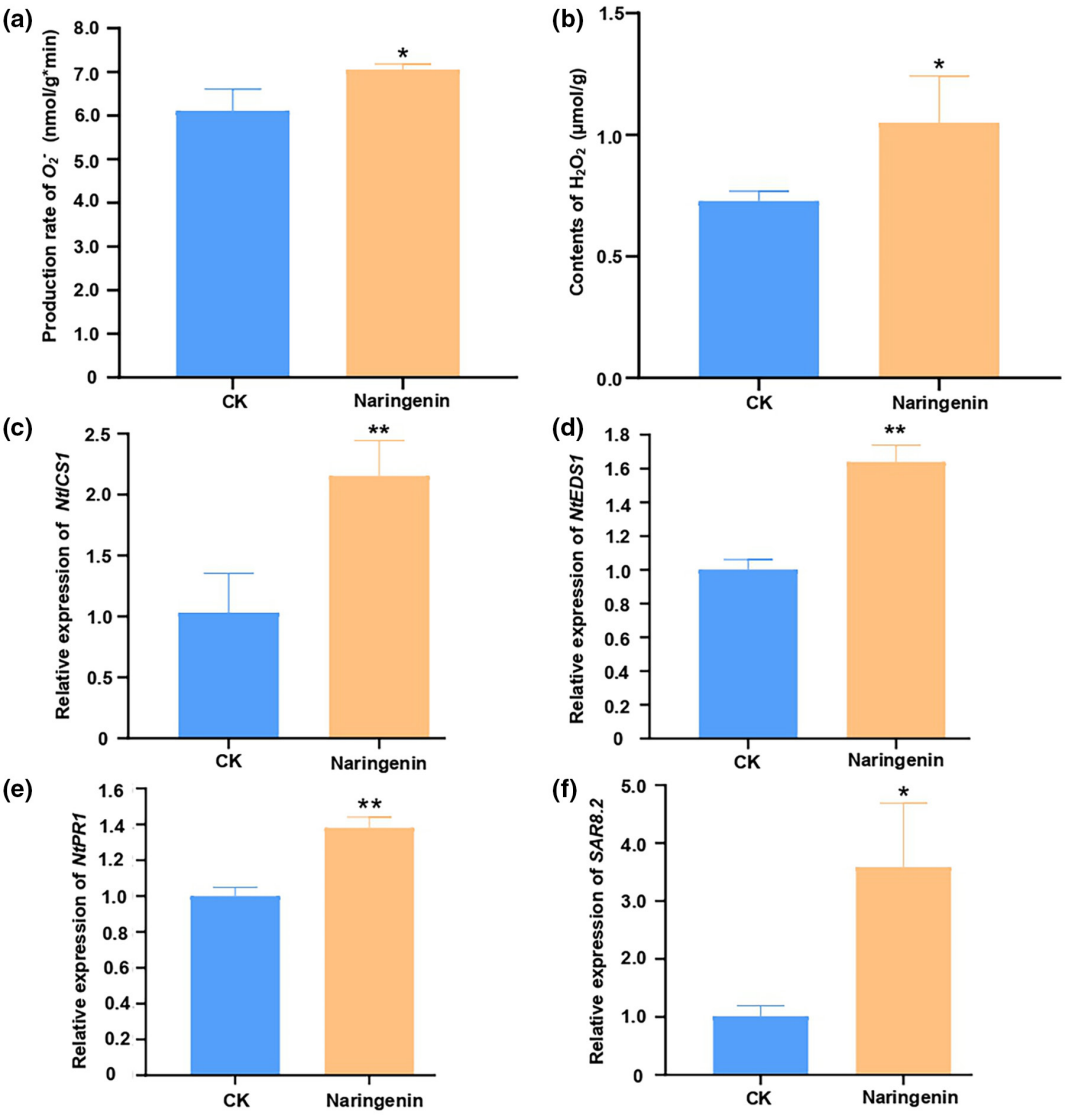
Naringenin induced plant resistance against *Phytophthora nicotianae* in Honghuadajinyuan (HD), the main flue‐cured tobacco variety, which is susceptible to tobacco black shank. Control (CK), the seedlings were treated without naringenin. (a) Statistical analysis for the rate of production of O_2_^−^ with or without treatment with naringenin 3 days postinoculation with *P. nicotianae* (*n* = 3, error bars = *SD*). (b) Statistical analysis for the content of H_2_O_2_ with or without treatment with naringenin 3 days postinoculation with *P. nicotianae* (*n* = 3, error bars = *SD*). (c) Relative expression levels of *NtICS1* after treatment with naringenin (three biologically independent experiments, each with three technical replicates). (d) Relative expression levels of *NtEDS1* after treatment with naringenin (three biologically independent experiments, each with three technical replicates). (e) Relative expression levels of *NtPR1* in HD after treatment with naringenin (three biologically independent experiments, each with three technical replicates). (f) Relative expression levels of *SAR8.2* after treatment with naringenin (three biologically independent experiments, each with three technical replicates). Significant differences compared to the control by Student's *t* test (*p < 0.05, **p < 0.01).

Normally, plants are resistant to the invasion of most pathogens through a burst of reactive oxygen species (ROS), including hydrogen peroxide (H_2_O_2_) and the superoxide anion (O_2_^−^) (Bray, 2000). The increased H_2_O_2_ content promotes the up‐regulation of genes that are associated with the plant defence response (Bray, 2000). Flavonoids have been shown to be antioxidant agents by clearing away ROS (Turek & Stintzing, 2013). Quercetin, a flavonoid and powerful antioxidant, has been reported to activate *Arabidopsis* defence against *Pseudomonas syringae* pv. *tomato* DC3000 via an H_2_O_2_ burst (Jia et al., 2010). To determine if ROS accumulate in tobacco with naringenin treatment, we tested the content of H_2_O_2_ and O_2_^−^ in Honghuadajinyuan (HD) seedlings (Figure 3a,b). HD is an important flue‐cured tobacco variety that is susceptible to TBS. As expected, the accumulation of H_2_O_2_ and O_2_^−^ was significantly induced with naringenin treatment, indicating that naringenin induced an ROS burst in tobacco.

### 2.4 Naringenin induced expression of salicylic acid biosynthesis‐related genes and signalling in tobacco

The hormone salicylic acid (SA) is required for the activation of immune responses to biotrophic pathogens in plants (van Butselaar & Van den Ackerveken, 2020). Various flavonoids mediate the activation of plant‐pathogen resistance by the SA‐dependent pathway (Jia et al., 2010; Yang et al., 2016). To test whether SA biosynthesis‐related genes could be induced by naringenin, the expression of the representative SA biosynthesis‐related gene *ICS1* was measured by RT‐qPCR. The expression of the *ICS1* gene increased approximately 2‐fold with naringenin treatment (Figure 3c). This result suggests that naringenin induces an increase in SA biosynthesis by the isochorismate‐dependent pathway and prompted us to examine whether naringenin induces SA signalling. We measured the expression of the SA signalling gene *EDS1* after treatment with naringenin by RT‐qPCR. The expression of *EDS1* was increased 1.8‐fold by naringenin treatment (Figure 3d). This result indicates that naringenin induces SA biosynthesis and activates SA signalling in tobacco.

### 2.5 Naringenin enhanced plant resistance to *P*. *nicotianae*

Flavonoids have been shown to be critical to plant defence on pathogenic bacteria and fungi through the induction of *PR* genes (Mierziak et al., 2014). Quercetin and its derivatives are capable of inducing pathogen resistance to both bacteria and fungi (Jia et al., 2010; Parvez et al., 2004; Yang et al., 2016). To examine whether naringenin induces basal pathogen resistance, we analysed the expression of *PR1* and *SAR8.2* genes in tobacco after naringenin treatment using RT‐qPCR. The *SAR8.2* gene is a gene that controls plant resistance to *P*. *nicotianae* (Shi et al., 2022). As expected, the expression levels of *PR1* and *SAR8.2* with naringenin treatment were approximately 1.4‐fold and 3.5‐fold higher than those of the control, respectively (Figure 3e,f). These results suggest that naringenin induces basal pathogen resistance.

### 2.6 Interference of naringenin biosynthesis led to more susceptibility to *P*. *nicotianae in tobacco*

**FIGURE 4:**
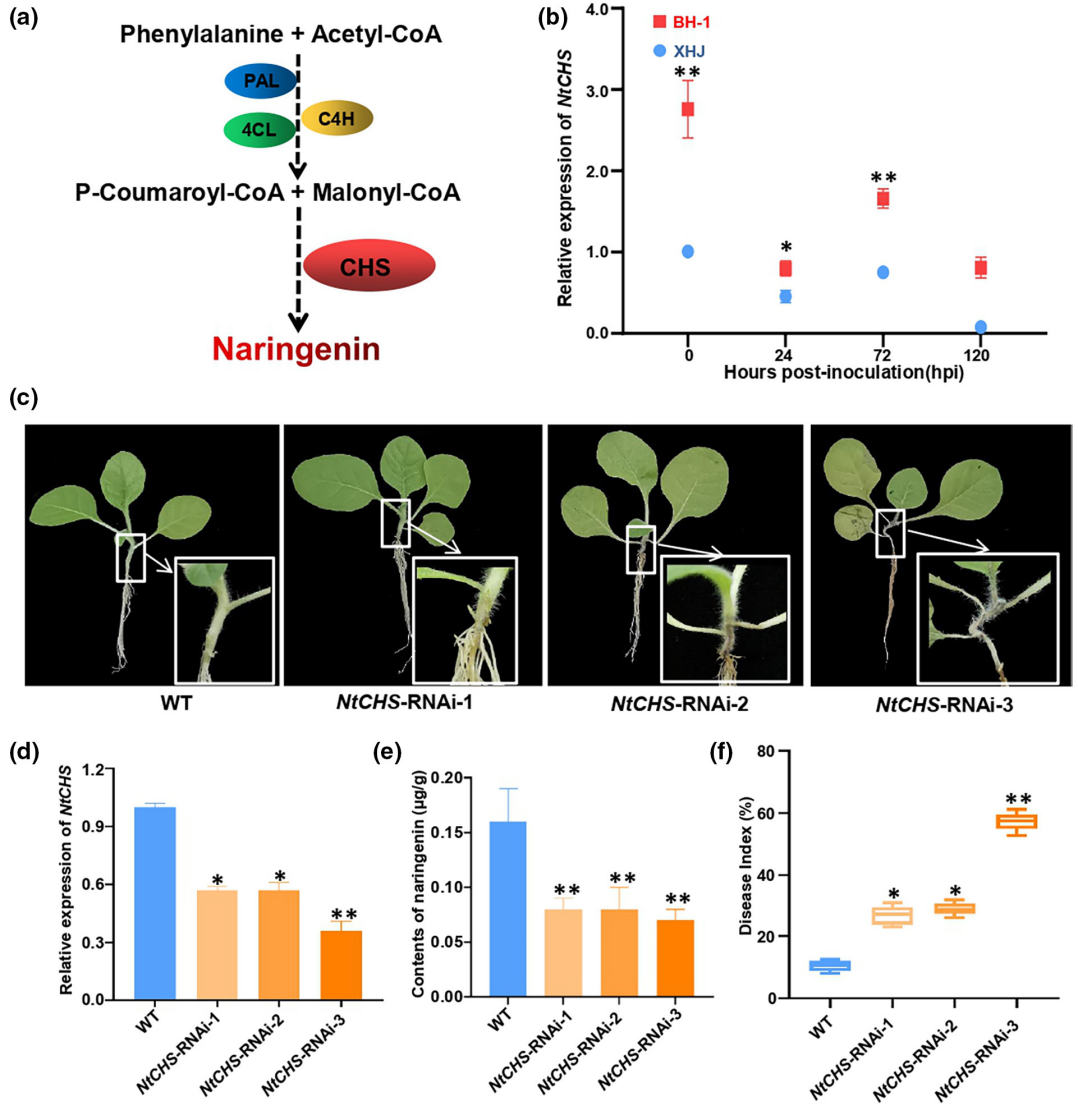
Transgenic validation of *NtCHS* conferring resistance to *Phytophthora nicotianae* in tobacco. (a) The pathway of naringenin synthesis. (b) Reverse transcription‐quantitative PCR analysis of *NtCHS* expression. (c) Disease symptoms of the T_2_ progeny derived from three transformants (*NtCHS*‐RNAi‐1, *NtCHS*‐RNAi‐2 and *NtCHS*‐RNAi‐3) obtained from transformation of wild‐type (WT) tobacco with an ubi::Tachs RNAi construct at 5 days postinoculation (dpi). (d) Relative *NtCHS* expression levels detected in the T_2_ progenies derived from the three RNAi transformants (three biologically independent experiments, each with three technical replicates). (e) RNAi of *NtCHS* decreased naringenin levels in the entire plant. (f) Disease index of the T_2_ progenies derived from three transformants (*NtCHS*‐RNAi‐1, *NtCHS*‐RNAi‐2 and *NtCHS*‐RNAi‐3) 5 dpi. (b, d–f) (*n* = 3, error bars = *SD*). * Significant differences compared to WT by the Mann–Whitney test (*p < 0.05, **p < 0.01).

The flavonoid biosynthesis pathway has been well studied and most intermediate enzyme steps have been characterized (see Figure 4a). The pathway starts with the conversion of phenylalanine to cinnamic acid by phenylalanine ammonia‐lyase (PAL). Under continuous catalysis by cinnamate 4‐hydroxylase (C4H) and 4‐coumaroyl coenzyme A (CoA) ligase (4CL), cinnamic acid is converted to *p*‐coumarinyl CoA, a substrate of flavonoids. Decarboxylative condensation of *p*‐coumarinyl CoA with three molecules of malonyl CoA into naringenin chalcones by chalcone synthase (CHS) provides substrates for the final synthesis of naringenin by chalcone isomerase (CHI) (Kreuzaler & Hahlbrock, 1972; Pandey et al., 2015). We first tested the expression of *NtCHS*, *NtPAL*, *NtC4H* and *Nt4CL* in BH‐1 and XHJ without and with inoculation of *P*. *nicotianae*. We found that, compared to XHJ, *NtCHS* was more highly expressed in BH‐1 even when infected with *P*. *nicotianae*, indicating that *NtCHS* might be a core regulatory gene in the biosynthesis of naringenin in tobacco (Figure 4b). The other three genes (*NtPAL*, *NtC4H* and *Nt4CL*) involved in the synthesis of naringenin did not show significant differences except in the later period (5 days) after tobacco was inoculated with *P*. *nicotianae* (Figure S5).

Next, to interfere with the naringenin synthesis pathway, we generated tobacco transgenic lines with *NtCHS* silenced via RNA interference (RNAi) in the resistant cultivar BH‐1 (Figure 4c). Three independent T_2_ transgenic lines comprising the *NtCHS* RNAi construct showed a significant decrease in the expression level of *NtCHS* (Figure 4d) and a dramatic decrease in naringenin content compared to the wild type (WT) (Figure 4e). These transgenic lines showed more susceptibility to *P*. *nicotianae* compared to the WT, but they kept the same sensitivity to *Ralstonia solanacearum*, a serious pathogen that causes tobacco bacterial wilt (Figures 4f and S6). Naringenin showed very low antimicrobial activity against *R. solanacearum* even at concentrations up to 400 mg/L (Table S7). Furthermore, the biomass of *P*. *nicotianae* in *NtCHS‐*RNAi mutants was up to six times more than that in WT by DNA quantification of the oomycete (Figure S7). These results show that interference in naringenin synthesis in tobacco induced more susceptibility to *P*. *nicotianae* and naringenin might be a specific antimicrobial agent to *P*. *nicotianae* because it showed very low or no activity towards other plant pathogens.

### 2.7 Exogenous application of naringenin enhanced resistance to *P*. *nicotianae*

**FIGURE 5:**
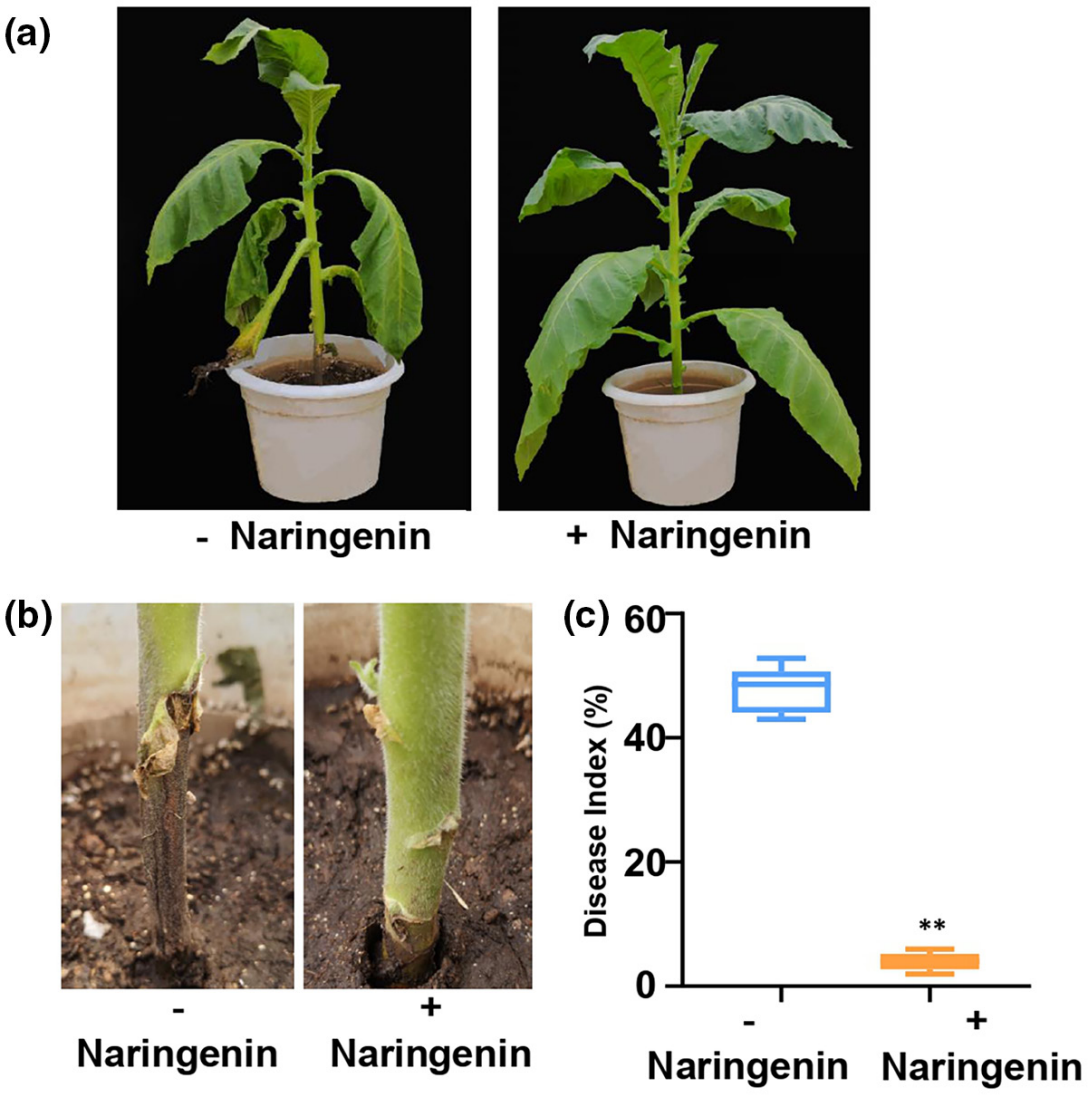
Resistance to tobacco black shank was enhanced by exogenous application of naringenin. (a) Disease symptoms of Honghuadajinyuan (HD) supplied with 100 ml of a solution of 0.4 g/L naringenin 10 days postinoculation with *Phytophthora nicotianae*. HD was inoculated with *P. nicotianae* but without treatment of naringenin as a control. (b) Enlarged view of the neck with or without treatment of naringenin after HD was inoculated with *P. nicotianae*. (c) The disease index with or without the treatment of naringenin after HD was inoculated with *P. nicotianae* (*n* = 3, error bars = *SD*). HD is the main cultivated flue‐cured tobacco variety but is susceptible to tobacco black shank. Significant difference compared to control by the Mann–Whitney test (**p < 0.01).

To explore the possibility of applying naringenin as a natural antimicrobial agent in the management of TBS in the field, we treated susceptible tobacco HD seedlings with naringenin before they were inoculated with *P*. *nicotianae*. HD seedlings inoculated with *P*. *nicotianae* but without naringenin treatment were used as the control. After 10 days, we found that HD seedlings treated with naringenin were significantly more resistant to *P*. *nicotianae* compared to the control (Figure 5a,b). Accordingly, the disease index in HD treated with naringenin was dramatically lower than in the control (Figure 5c). More importantly, the application of naringenin did not negatively affect the agronomic traits of tobacco, such as plant height, leaf length, leaf width, knot spacing and stem girth (Figure S8). In addition, to test the impact of naringenin stability on infection by pathogens, we checked the susceptibility of HD tobacco to *P*. *nicotianae* 3, 7, 15 and 30 days after treatment with naringenin. The results showed that HD treated with naringenin for 30 days still had the same susceptibility to *P*. *nicotianae* as HD treated with naringenin for 3 days, and both were significantly more resistant to *P*. *nicotianae* compared to HD without naringenin treatment. These results show that exogenous application of naringenin improved resistance to *P*. *nicotianae* significantly and showed good stability of antimicrobial activity in the environment (Figure S9). Therefore, naringenin was shown to be a potential antimicrobial agent for the management of TBS in the field.

## 3 DISCUSSION

Plants have evolved diverse biosynthetic routes to produce a range of small organic molecules referred to as secondary metabolites. Secondary metabolites are restricted to specific taxonomic groups and play important roles in diverse aspects of plant life activities, including as components of signalling cascades and in plant defence against herbivores (Pandey et al., 2015). Among all plant secondary metabolites, flavonoids are widely studied as they have a multitude of biological functions, including a function as defence molecules against biotic and abiotic stresses (Treutter, 2005; Zhang et al., 2020). Flavanones and flavonols are categories of flavonoids that differ by a hydroxyl moiety in the 3′ position in flavonols that is lacking in flavanones (Sun et al., 2019). Flavanones include phloretin, glyceollins and naringenin. Aminoethyl‐phloretin is a water‐soluble phloretin derivative that has been shown to possess antibacterial activity toward both gram‐positive and gram‐negative bacteria (Wei et al., 2020). Glyceollins are a group of phytoalexins that are mainly isolated from soybeans. Both aminoethyl‐phloretin and glyceollins have numerous functions in human health, especially as anticancer agents (Pham et al., 2019). Naringenin is a typical flavanone that has been found mostly in edible fruits such as citrus species, tomatoes and figs, and studies on naringenin have mainly focused on its biological effects on human health (Salehi et al., 2019). There are a few reports on naringenin and its antimicrobial activity on *Escherichia coli* O157:H7 in apple cider and its improvement of resistance to rice blast (Surendran Nair et al., 2020). However, there are no reports on the mechanism by which naringenin provides defence against plant pathogens.

In this study, we found that there was a significant difference in the basal flavonoid metabolite profile between resistant and susceptible tobacco varieties. We treated *P*. *nicotianae* in vitro with different concentrations of flavanones and flavonols, and found that only three flavanones showed antimicrobial activity, with naringenin showing the highest activity. However, none of the flavonols showed any antimicrobial activity, even at high concentrations up to 200 mg/L. Compared to its activity against *P*. *nicotianae*, naringenin had a lower antimicrobial activity against other oomycetes such as  *P. capsici*. Naringenin showed no or very low inhibitory activity towards plant pathogens such as *S. sclerotiorum* and *B. cinerea*. Interestingly, there was dramatic inhibition of sporangia production when *P*. *nicotianae* was treated with a very low concentration of naringenin. The reason for the inhibition of sporangia production by naringenin might be that genes regulating the reproductive process in *P*. *nicotianae* are directly suppressed by naringenin. We used RT‐qPCR to confirm that genes regulating cell growth and reproduction were down‐regulated after *P*. *nicotianae* was treated with naringenin. Naringenin is thus the first flavonoid to be identified as a antimicrobial agent against *P*. *nicotianae;* it might affect the expression of genes regulating the growth and reproduction of *P*. *nicotianae* directly.

Flavonoids are well known as ROS scavengers and powerful antioxidants (Pannala et al., 2001; Rice‐Evans, 2001). Flavonoids decrease the ROS level through inhibition of pro‐oxidant enzymes, including cyclo‐oxygenase and lipoxygenase (Eghbaliferiz & Iranshahi, 2016). Phenolics and carotenoids are chemical groups that prevent oxidative damage as a result of their ability to decrease the ROS level, and they also exhibit pro‐oxidant activities in vitro in the presence of metal ions (Eghbaliferiz & Iranshahi, 2016). Like phenolics and carotenoids, flavonoids act as pro‐oxidants at physiological pH. In this study, we showed that naringenin induced the accumulation of ROS, which suggests that naringenin might act as a pro‐oxidant. An et al. also showed that naringenin induced an ROS burst in plants as a defence against *P. syringae* (An et al., 2021). SA has been shown to induce pathogen resistance through ROS accumulation and increased expression of *PR* genes. We also found that genes regulating SA biosynthesis, SA signalling and basal pathogen resistance were enhanced at the transcriptional level by treatment with naringenin . Furthermore, *SAR8.2*, a gene in tobacco that regulates plant resistance to *P*. *nicotianae* (Shi et al., 2022), was substantially up‐regulated by treatment with naringenin. These results indicate that naringenin induces plant‐pathogen resistance. Naringenin accumulates soon after plants are infected with biotrophic pathogens such as *Rhizobium leguminosarum* bv. *viceae*, *Pseudomonas syringae* pv. *pisi* and *Plasmodiophora brassicae* (Makarova et al., 2016). However, we did not detect significant accumulation of naringenin in tobacco after infection with *P*. *nicotianae* by metabolite analysis. Interestingly, naringenin has been shown to induce pathogen resistance against *P. syringae* through the activation of *NPR1* in *Arabidopsis* 2021, but did not show any antimicrobial activity against *P. syringae* even at high concentrations in vitro (An et al., 2021). Our results demonstrate that naringenin confers plant defence against *P*. *nicotianae* through induction of plant‐pathogen resistance in addition to its antimicrobial activity.

The key genes in the metabolic pathway of naringenin synthesis include *PAL*, *4CL*, *C4H*, and *CHS* (Kreuzaler & Hahlbrock, 1972; Pandey et al., 2015). Our results showed that the expression level of *NtPAL*, *NtC4H* and *Nt4CL* did not show a significant change except in the later period (5 days) after BH‐1 had been infected by *P*. *nicotianae* (Figure S5). Remarkably, only *NtCHS* had a higher expression level in BH‐1 than that in XHJ, even after BH‐1 was treated with *P*. *nicotianae*. The accummulation of naringenin is positively correlated with the expression of *CHS* (Pandith et al., 2019). In this study, without infection of *P*. *nicotianae*, the basal expression level of *NtCHS* in resistant tobacco variety BH‐1 was higher than that in susceptible tobacco variety XHJ. Correspondingly, the content of naringenin in BH‐1 was higher than that in XHJ. CHS is a rate‐limiting enzyme that controls the supply of substrates in the flavonoid biosynthesis pathway, thus the amount of products downstream are affected by the efficiency of CHS. Naringenin plays a key role in both plant‐pathogen resistance and antimicrobial activity after the plant is infected by *P*. *nicotianae;* however, the plant is not able to synthesize  unlimited amounts of naringenin as there is a lack of substrates in the biosynthesis pathway. The expression level of *NtCHS* was down‐regulated to keep the flavonoid biosynthesis pathway in order (Figure 4b). RNA interference of *NtCHS* led to a dramatic decrease in naringenin content and the biomass of *P*. *nicotianae* in *NtCHS‐*RNAi tobacco mutants was up to 6‐fold higher than that in WT plants. The level of RNA interference in different RNAi transgenic plant lines might vary. In this study, compared to the other two RNAi transgenic lines, the content of naringenin in the *NtCHS*‐RNAi‐3 transgenic line was much less and this may be the reason that the *NtCHS*‐RNAi‐3 transgenic line was more susceptible to *P. nicotianae*. Significantly, TBS severity is a quantitative trait and its phenotype might be affected by multiple factors in the environment. Therefore, genes that regulate the plant resistance to TBS and the plant phenotype do not  correspond to each other, l, raising difficulties to study the mechanism of plant resistance to TBS.

*CHS* orchestrates the general flavonoid biosynthesis pathway in tobacco; thus, its down‐regulation influences the biosynthesis of all chalcones, flavones, anthocyanins and other derivatives and is not limited to naringenin. In fact, the accumulation of various flavonoids, including naringenin and rutin, decreases significantly in *NtCHS1*‐RNAi transgenic plants (Chen et al., 2019). However, only naringenin showed antimicrobial activity against *P*. *nicotianae*, and other flavonoids such as rutin showed very low or no antimicrobial activity. By contrast, compared to the WT, we found that the *NtCHS‐*RNAi tobacco mutants kept the same susceptibility to *R. solanacearum*, a serious pathogen which causes tobacco bacterial wilt. This indicates that RNAi of *NtCHS* induced a reduction in the content of naringenin, and specific antimicrobial activity against *P*. *nicotianae* by naringenin might be the main reason that *NtCHS‐*RNAi tobacco was more sensitive to *P*. *nicotianae* compared to other plant pathogens such as *R. solanacearum*. Naringenin showed no antimicrobial activity against *R. solanacearum* in vitro even with concentrations of naringenin up to 400 mg/L. Because naringenin induced resistance against both *P*. *nicotianae* and *P. syringae*, naringenin could also be expected to induce resistance to *R. solanacearum*. *CHS* regulates flower colour, fertility and gas substances, and we found that *CHS* was also involved in resistance to *P*. *nicotianae*. We also found that TBS‐susceptible tobacco variety HD became resistant to *P*. *nicotianae* after treatment with naringenin. Naringenin showed high activity with a good stability in the environment (Figure S9). This leads to the potential application of naringenin as a natural antimicrobial agent in the management of TBS in the field in the future.

In conclusion, for first time we found that naringenin might be an antimicrobial secondary metabolite against *P*. *nicotianae*. Naringenin inhibited *P*. *nicotianae* through not only its antimicrobial activity, but also its induction of defences against plant pathogens (Figure S10). Our discoveries will enrich the means of prevention and control for TBS. Future work needs to address the question of whether naringenin targets proteins or genes in *P*. *nicotianae* directly, and whether naringenin interferes in the interaction between *P*. *nicotianae* and tobacco. Notably, the development of naringenin as a plant‐derived fungicide against *P*. *nicotianae* is important work for the future.

## 4 EXPERIMENTAL PROCEDURES

### 4.1 Plant materials and *P. nicotianae* inoculation

BH‐1 shows a high level of resistance to TBS, whereas XHJ is extremely susceptible. Both of these were obtained from the Tobacco Research Institute, Chinese Academy of Agricultural Sciences. Plants were cultured using Holland nutrient solution and were grown in a growth chamber at 28°C with 16 h light and 8 h dark photoperiod cycles. Sixteen‐week‐old tobacco plants were infected with *P. nicotianae* by dipping tobacco in a spore suspension for 3 h, then cultured in water. After 5 days, the phenotype of TBS was evaluated using an empirical scale (YC/T39‐1996, China), where 0 represents a highly resistant response and 9 represents a highly susceptible response. Disease index scores based on disease severity were used for assessment and calculated using the following formula: disease index (%) = [∑(disease evaluation scale score × number of plants with each scale score)/(total number of plants observed × the highest disease evaluation scale score)] × 100. Three‐month‐old tobacco plants were infected with *P*. *nicotianae* according to a described method (Zhang et al., 2018). The biomass of *P*. *nicotianae* in infected tobacco plants was quantified using a slight modification of a previously described method (Park et al., 2012). Quantitative PCR was performed using a Light Cycler 96 Real‐Time PCR detection system (Roche, http://technical‐support.roche.com). The biomass of *P. nicotianae* was calculated using the threshold cycle value (*C*_t_) of *P. nicotianae* WS21 DNA against the *C*_t_ of tobacco genomic *actin* DNA.

### 4.2 Inoculation of tobacco with *R. solanacearum*

After 12‐week‐old tobacco plants were infected with *R. solanacearum*, a small amount of bacteria was picked up with a toothpick and placed into 1 L of nutrient broth (NB) (Qingdao Hope Bio‐Technology Co., Ltd). The bacteria were grown in an incubator at 30°C and 200 rpm until the OD_600_ reached 1.0. Two hundred millilitres of suspension bacteria cells was added to each plate of tobacco seedlings. After 15 days, the phenotype of tobacco bacterial wilt was evaluated using an empirical five‐point scale (GB/T 23222–2008), where 0 represents a highly resistant response and 4 represents a highly susceptible response. Disease index scores based on the disease severity were used for assessment and were calculated using the following formula: disease index (%) = [∑(disease evaluation scale score × number of plants with each scale score)/(total number of plants observed × the highest disease evaluation scale score)] × 100.

### 4.3 Pathogen isolation and inoculum preparation

*R. solanacearum* and *P. nicotianae*  race 0 were obtained from the Plant Protection Laboratory of the Chinese Academy of Agricultural Sciences. *P. capsici*, *P. aphanidermatum*, *Pythium ultimum*, *S. sclerotiorum*, *Colletotrichum gloeosporioides*, *Fusarium moniliforme*, *B. cinerea*, and *F. graminearum* were obtained from Nanjing Agricultural University. Each pathogen was grown separately in 25 ml of potato dextrose agar (PDA; potato 200 g, glucose 20 g, agar 16 g, in 1 L) at 28°C for 14 days. The inoculum method was used as described (Zhang et al., 2018). Then 0.1% KNO_3_ was added to the PDA plate containing *P. nicotianae* and kept at 4°C for 25 min, then kept in the light for 30 min at 25°C. The concentration of the spore suspension was determined by a cellometer Auto T4 (Nexcelom Cellometer) and adjusted to 10^4^ spores/ml.

### 4.4 Antimicrobial activity test for flavonoids

All flavonoid standards used in this study were purchased from the Solarbio Company. First, 20 mg of standard flavonoid was dissolved in 1 ml of ethanol to make 20 mg/ml stock solution, which was filtered and sterilized before use. PDA containing different concentrations of different flavonoids was prepared for the antimicrobial tests. A single agar plug (5 mm diameter) was removed from the actively growing edge of the fungal culture and placed in a new PDA culture containing flavonoids. Four different pathogens inoculated with PDA or with only ethanol added served as positive controls. The medium was observed after incubating at 28°C for 7 days. The efficacy of each treatment was evaluated by measuring the diameter of each colony. Each treatment contained three replicates. The inhibition rate was calculated as follows: inhibition rate (%) = (control hyphae diameter − treatment hyphae diameter)/control hyphae diameter × 100.

### 4.5 Antimicrobial activity of naringenin on *R. solancearum*

First, 0.1 ml of 10^8^ cfu/ml *R. solancearum* suspension was pipetted onto a Petri dish (diameter of 9 cm). Then the cooled and melted PDA medium was mixed thoroughly with the *R. solancearum* suspension. Filter paper was punched into 6‐mm diameter discs with a hole punch, immersed in different concentrations of naringenin, then moved to a flat plate. The plates were placed in an incubator at 28°C for 3 days. The diameter of the bacteriostatic circle was measured by the cross‐crossing method, and the average weight of each treatment was calculated. Each treatment contained three replicates. The inhibition rate was calculated as follows: inhibition rate (%) = (treatment bacteriostatic circle − control bacteriostatic circle)/control bacteriostatic circle × 100.

### 4.6 Metabolite estimation and analysis

Eight‐week‐old seedlings of BH‐1 and XHJ grown in Hoagland hydroponic nutrient solution were used for flavonoid metabolomic analysis. The freeze‐dried leaves were crushed using a mixer mill (MM 400; Retsch) with a zirconia bead for 1.5 min at 30 Hz. One hundred milligrams of powder extracted overnight at 4°C with 1 ml of 70% methanol. Following centrifugation at 10,000 × *g* for 10 min, the extracts were absorbed and filtrated (SCAA‐104, 0.22 μm pore size) before LC‐ESI‐MS/MS (HPLC, Shim‐pack UFLC SHIMADZU CBM30A system; MS, Applied Biosystems 4500 Q TRAP) analysis. The sample extracts were analysed by LC‐ESI‐MS/MS according to methods described previously (Gong et al., 2013). The effluent was connected to an ESI‐triple quadrupole‐linear ion trap (QTRAP)‐MS. Linear ion trap (LIT) and triple quadrupole (QQQ) scans were acquired on a triple quadrupole‐linear ion trap mass spectrometer (Q TRAP; API 4500 Q TRAP LC/MS/MS System) equipped with an ESI Turbo ion‐spray interface operating in a positive ion mode and controlled by Analyst v. 1.6.3 software (AB Sciex). The ESI source operation parameters were as follows: ion source, turbo spray; source temperature 550°C; ion spray voltage (IS) 5500 V; ion source gas I (GSI), gas II (GSII), curtain gas (CUR) set at 55, 60 and 25.0 psi, respectively; collision gas (CAD) high. Instrument tuning and mass calibration were performed with 10 and 100 μM polypropylene glycol solutions in QQQ and LIT modes, respectively. QQQ scans were acquired as multiple reaction monitoring (MRM) experiments with collision gas (nitrogen) set to 5 psi. DP (declustering potential) and CE (collision energy) for individual MRM transitions was done with further optimization of DP and CE. A specific set of MRM transitions was monitored for each period according to the metabolites eluted within this period.

Based on the self‐built database MWDB (Metware database) and the public database of metabolite information, qualitative analysis was performed on the primary and secondary mass spectrometry data. Metabolite quantification was carried out via the mode of MRM. After obtaining metabolite spectrum analysis data for different samples, peak area integration was performed on the mass spectrum peaks of all substances and integration correction was performed on the mass spectrum peaks of the same metabolite in different samples. To screen out the differential metabolites between the BH‐1 and XHJ groups, the mass spectrum peak of each metabolite in each sample was corrected according to the retention time and peak shape of each metabolite, thereby ensuring the accuracy of the qualitative and quantitative analyses. Supervised multiple regression orthogonal partial least‐squares discriminant analysis (OPLS‐DA) was conducted to estimate the stability and reliability of the model. The threshold variable importance in projection (VIP) value ≥1 and fold change ≥2 (up‐regulated) or ≤0.5 (down‐regulated) were used for screening the differential metabolites and drawing the heatmap.

### 4.7 RNA extraction of plants, cDNA synthesis and RT‐qPCR

Total RNA was extracted with a Plant RNA pure kit (ZOMANBIO, ZP405‐2), then the reverse transcription of the cDNA was synthetized with HiScript III RT SuperMix for qPCR (Vazyme). The primers for amplification of all genes were designed by Primer Premier v. 5 software. The qPCR was performed by a LightCycler 96 Real‐Time PCR detection system (Roche, http://technical‐support.roche.com) using ChamQ SYBR Colour qPCR Master Mix (Vazyme, Q411‐02/03). The relative expression levels of genes were calculated by the 2^−ΔΔ*C*t^ method and all reactions were repeated three times. Three independent biological replicates were used. The sequences of all primers are shown in Table S8.

### 4.8 The production of *NtCHS*‐RNAi lines

The construction of NtCHS‐RNAi and generation of transgenic tobacco lines have been described previously (Chen et al., 2019). PCR identification was used to amplify the transformed seedling DNA with RNAi‐F and RNAi‐R primers to determine whether the RNAi vector was successfully introduced. For the transformed lines, the silencing efficiency of *NtCHS* was identified by RT‐qPCR. The sequences for all primers used in this experiment are shown in Table S8.

### 4.9 Naringenin measurement by LC‐ESI‐MS/MS

Eight‐week‐old tobacco plants of WT and *NtCHS*‐RNAi lines were sampled and ground into homogenate using liquid nitrogen. After freeze‐drying, the 20‐mg samples were suspended in 1 ml of extraction solution (methanol:chloroform:water = 5:2:2) and treated by ultrasonic vibration  for 45 min to extract naringenin. The extracted naringenin was analysed using LC‐ESI‐MS/MS (HPLC, Shim‐pack UFLC Shimadzu CBM‐20A system; MS, Applied Biosystems 4000 Q TRAP) according to the methods described previously (Zhao et al., 2020). Three biological replicates of each treatment were performed. The chromatographic conditions were as follows: Waters BEH C18 chromatographic column (150 mm × 2.1 mm, 1.7 μm); mobile phase: A phase water, B phase acetonitrile, 0.1% (vol/vol) formic acid and 0.2 mM ammonium acetate were added t both phases. Step‐stripping sequence: 0–1.0 min, 10% B; 0–9.0 min, 10% B–90% B; 9.0–11.0 min, 90% B–100% B; 11.0–11.1 min, 100% B–10% B; 11.1–13.0 min, 10% B. Column temperature: 30°C; injection volume: 2 μl; flow rate: 0.25 ml/min. Mass spectrum conditions: retention time 4.94 min^−1^; ion pair l: quantitative ion 433.1/271.1, collision energy −22 V; ion pair 2: quantitative ion 433.1/150.9, collision energy −42 V.

### 4.10 Inhibition of sporangia production

The effect of different concentrations of naringenin on sporangia production of *P*. *nicotianae* was assayed. A fresh mycelial block was immersed in V8 liquid medium and cultivated at 28°C for 48 h. Mycelium was washed with sterile deionized water, then soaked in sterile deionized water containing 0, 0.78, 1.56, 3.13, 6.25, and 12.50 mg/L of naringenin. Following a 24‐h incubation period at 28°C in the light, the sporangia were counted by observation with a microscope using three fields. Each treatment contained three replicates. The inhibition rate was calculated as follows: inhibition rate (%) = (control number of sporangia − treatment number of sporangia)/control number of sporangia × 100.

### 4.11 Measurement of H_2_O_2_ content and production rate of O_2_ ^−^ content

Three days after inoculation with *P. nicotianae*, HD roots treated with naringenin were used to measure ROS levels. The H_2_O_2_ content was determined by measuring the yellow titanium peroxide compound, which had specific absorption peak at 415 nm, according to the instruction of H_2_O_2_ Content Detection Kit (Suzhou Keming Biotechnology Ltd Co.). The production rate of O_2_^−^ was determined by measuring the formation of red azo compound, which had a specific absorption peak at 530 nm, according to the O_2_^−^ Detection Kit (Suzhou Keming Biotechnology Ltd Co.).

### 4.12 Exogenous application of naringenin

Under simulated field conditions, *Nicotiana tabacum* ‘Honghuadajinyuan’ (HD) was cultured for 3 months in pots. Then, the stem base of the HD seedlings was treated with 100 ml of solution including 0.04 g of naringenin 1 week prior their inoculation with *P*. *nicotianae*. Naringenin was added only once. HD seedlings with inoculation by *P*. *nicotianae* but without prior treatment of naringenin were used as a negative control. After 10 days, the phenotype of TBS was evaluated using an empirical six‐point scale (YC/T39‐1996, China), where 0 represents a highly resistant response and 9 represents a highly susceptible response. Disease index scores based on disease severity were used for assessment and calculated using the following formula: disease index (%) = [∑(disease evaluation scale score × number of plants with each scale score)/(total number of plants observed × the highest disease evaluation scale score)] × 100.

## CONFLICT OF INTEREST

No conflict of interest declared.

## Supporting information


**Figure S1** The antimicrobial activity of the representative flavonols on *Phytophthora nicotianae*. The control (CK) was added ethanol only. (a) Colony morphology of *P. nicotianae* on potato dextrose agarose at 28°C for 7 days amended with kaempferol, quercetin or rutin. The concentrations were 12.5, 25, 50, 100 and 200 mg/L. (b) The inhibition rate of kaempferol, quercetin and rutin on *P. nicotianae*. (*n* = 3, error bars, *SD*).

**Figure S2** The antimicrobial activity of three other pathogens treated with naringenin. (a) Colony morphology of *Phytophthora capsici* on potato dextrose agarose (PDA) amended with a different concentration of naringenin at 28°C. The concentrations of naringenin were 12.5, 25, 50, 100, 200, and 400 mg/L. The control (CK) was added ethanol only. (b) Colony morphology of *Pythium aphanidermatum* and *Pythium ultimum* on PDA amended with different concentrations of naringenin at 28°C. The concentrations of naringenin were 25, 50, 75, 100, 125, 150 and 175 mg/L. The control (CK) was added ethanol only. (c,d) The inhibition rate of naringenin on *Phytophthora capsici*, *Pythium aphanidermatum* and *Pythium ultimum* (*n* = 3, error bars, *SD*).

**Figure S3** The inhibitory test of other phytopathogens treated with naringenin. The concentration of naringenin was 200 mg/L. The control (CK) was added ethanol only.

**Figure S4** The result of reverse transcription‐quantitative PCR (RT‐qPCR) validation for differentially expressed genes on cell growth and reproduction of *Phytophthora nicotianae*. The control (CK) means *P. nicotianae* was treated with solvent only. The NG means *P. nicotianae* was treated with 20 mg/L naringenin. Gene expression was determined by RT‐qPCR after sampling (*n* = 3, error bars, *SD*).

**Figure S5**Reverse transcription‐quantitative PCR (RT‐qPCR) analysis of *NtPAL*, *NtC4H* and *Nt4CL* expression levels. (a) RT‐qPCR analysis of *NtPAL*. (b) RT‐qPCR analysis of *NtC4H* expression levels. (c) RT‐qPCR analysis of *Nt4CL* expression levels (*n* = 3, error bars, *SD*). * indicates that gene expression level in BH‐1 has differences comparing to it in XHJ at 120 postinoculation (hpi) by Student’s *t* test (*p* < 0.05). ** indicates that gene expression level in BH‐1 has significant differences comparing to it in XHJ at 120 hpi by Student’s *t* test (*p* < 0.01).

**Figure S6** Disease index of the T_2_ progenies derived from three transformants (*NtCHS*‐RNAi‐1, *NtCHS*‐RNAi‐2 and *NtCHS*‐RNAi‐3) 5 days after inoculation of *Ralstonia solanacearum*. Disease index of the transgenic lines comparing to that of the wild type was showed as *p* values by Student’s *t* test.

**Figure S7** Biomass of *Phytophthora nicotianae* in the T_2_ progenies derived from three *NtCHS*‐RNAi transformants for 5 days after inoculation of *P. nicotianae*. *n* = 3, error bars, *SD*. * indicate that *NtCHS*‐RNAi‐1 and *NtCHS*‐RNAi‐2 has differences comparing to the wild type (WT) by Student’s *t* test (*p* < 0.05). ** indicates that *NtCHS*‐RNAi‐3 has significant differences comparing to WT by Student’s *t* test (*p* < 0.01).

**Figure S8** Agronomic traits of tobacco var. Honghuadajinyuan (HD) treated with naringenin. All agronomic trails were determined at 60 days after transplanting HD. (a) Plant height of HD treated with naringenin. (b) Leaf length of HD treated with naringenin. (c) Leaf width of HD treated with naringenin. (d) Knot spacing of HD treated with naringenin. (e) Stem girth of HD treated with naringenin. *n* = 6, error bars, *SD*. *p* values were calculated the transgenic lines comparing to the wild type by Student’s *t* test.

**Figure S9** The stability of naringenin. Honghuadajinyuan (HD) was inoculated with *Phytophthora nicotianae* after treated with a solution of 0.4 g/L naringenin for 3, 7, 15 and 30 days. HD not treated with naringenin was concurrently inoculated with *P. nicotianae* as a control. Statistical analysis for the disease index of HD with or without naringenin treatment postinoculation with *P. nicotianae* (*n* = 3, error bars, *SD*). * indicates that the desease index of HD treated with naringenin has differences compared to control by Mann–Whitney test (*p* < 0.05). ** indicates that the desease index of HD treated with naringenin has ignificant differences compared to control by Mann–Whitney test (*p* < 0.01).

**Figure S10** A model of how naringenin affects pathogen resistance to *Phytophthora nicotianae*. Once plants are under the attack from *P. nicotianae*, naringenin produced from plants will not only inhibit mycelial growth, but also sporangia production of *P. nicotianae*. Meanwhile, naringenin induced not only the accumulation of reactive oxygen species (ROS), but also salicylic acid (SA) induced basal plant pathogen resistance. Both antimicrobial activity and induction of plant defence by naringenin lead to plant resistance to *P. nicotianae*.

**Table S1** A total of 166 metabolites was characterized by their distinct retention times and mass‐to‐charge ratios (*m*/*z*)

**Table S2** Significant up‐regulated metabolites in Beinhart 1000‐1 (BH‐1) relative to Xiaohuangjin (XHJ)

**Table S3** A total of 16 flavanones were characterized in metabolite analysis

**Table S4** A total of 44 flavonols were characterized in metabolite analysis

**Table S5** The inhibitory activity of the different flavonoids on *Phytophthora nicotianae*

**Table S6** The inhibitory test of the three pathogens and sporangia production of *Phytophthora nicotianae* treated with naringenin

**Table S7** The inhibitory activity of naringenin on *Ralstonia solancearum*

**Table S8** All primer sequences used for this research

## Data Availability

The data that support the findings of this study are available from the corresponding author upon reasonable request.
