# Supplementary figures and images for: Naringenin confers defence against Phytophthora nicotianae through antimicrobial activity and induction of pathogen resistance in tobacco
Source: Mol Plant Pathol. 2022 Sep 12;23(12):1737–50. doi: 10.1111/mpp.13255 (PMC9644278; doi:10.1111/mpp.13255)

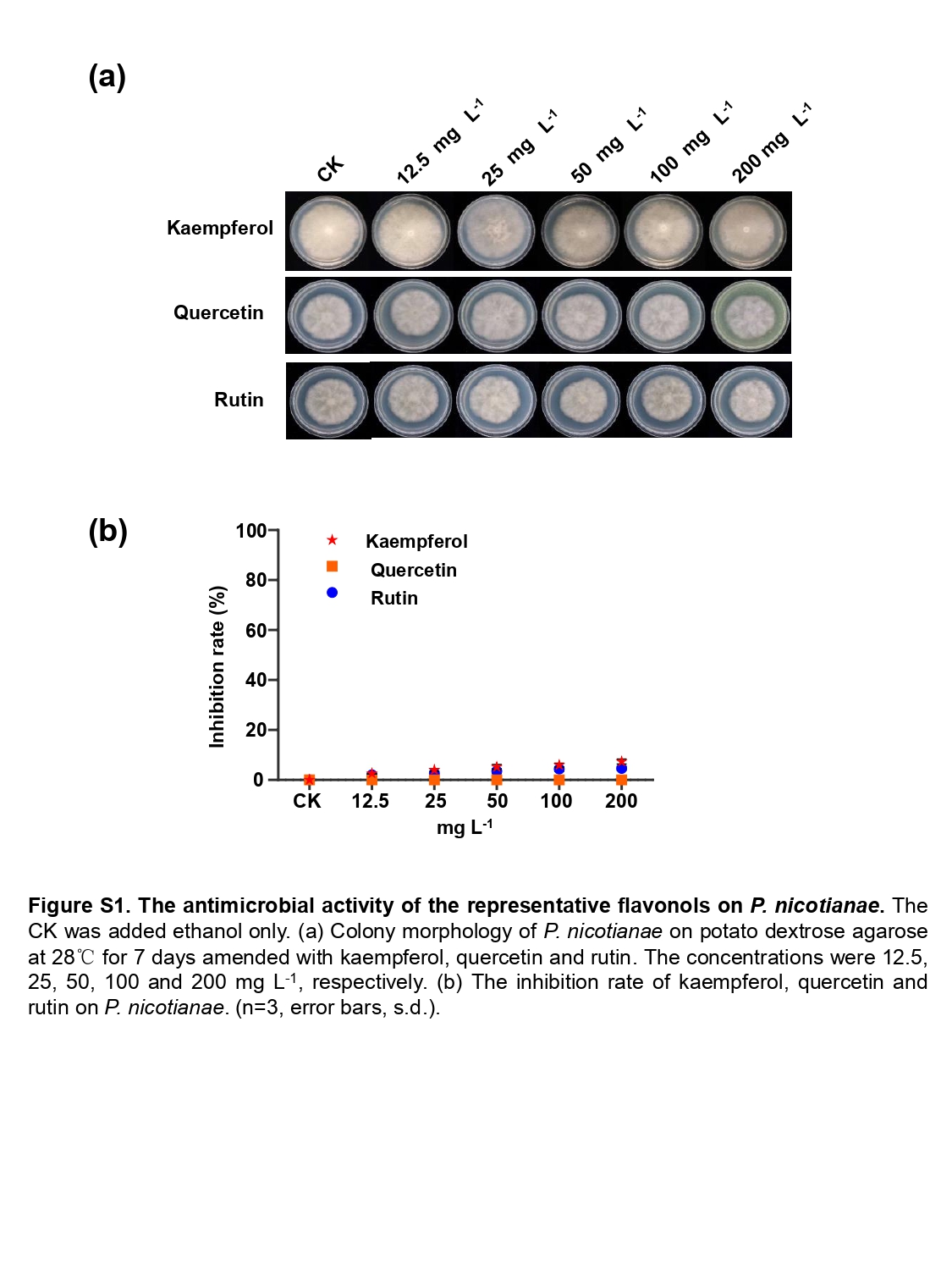

Supplement: Supplementary file 1 — Figure S1 The antimicrobial activity of the representative flavonols on Phytophthora nicotianae. The control (CK) was added ethanol only. (a) Colony morphology of P. nicotianae on potato dextrose agarose at 28°C for 7 days amended with kaempferol, quercetin or rutin. The concentrations were 12.5, 25, 50, 100 and 200 mg/L. (b) The inhibition rate of kaempferol, quercetin and rutin on P. nicotianae. (n = 3, error bars, SD). [file MPP-23-1737-s003.jpg]

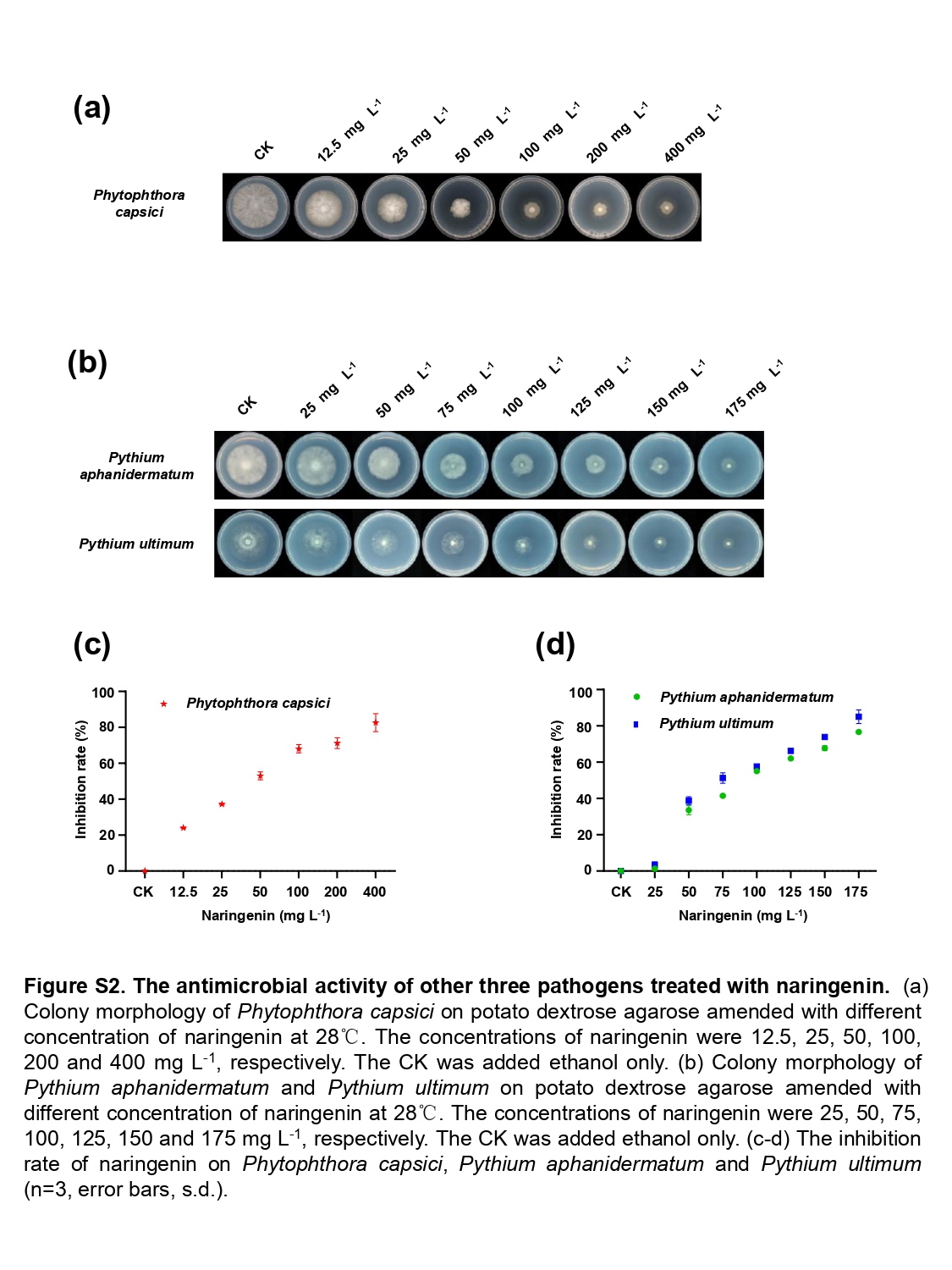

Supplement: Supplementary file 2 — Figure S2 The antimicrobial activity of three other pathogens treated with naringenin. (a) Colony morphology of Phytophthora capsici on potato dextrose agarose (PDA) amended with a different concentration of naringenin at 28°C. The concentrations of naringenin were 12.5, 25, 50, 100, 200, and 400 mg/L. The control (CK) was added ethanol only. (b) Colony morphology of Pythium aphanidermatum and Pythium ultimum on PDA amended with different concentrations of naringenin at 28°C. The concentrations of naringenin were 25, 50, 75, 100, 125, 150 and 175 mg/L. The control (CK) was added ethanol only. (c,d) The inhibition rate of naringenin on Phytophthora capsici, Pythium aphanidermatum and Pythium ultimum (n = 3, error bars, SD). [file MPP-23-1737-s017.jpg]

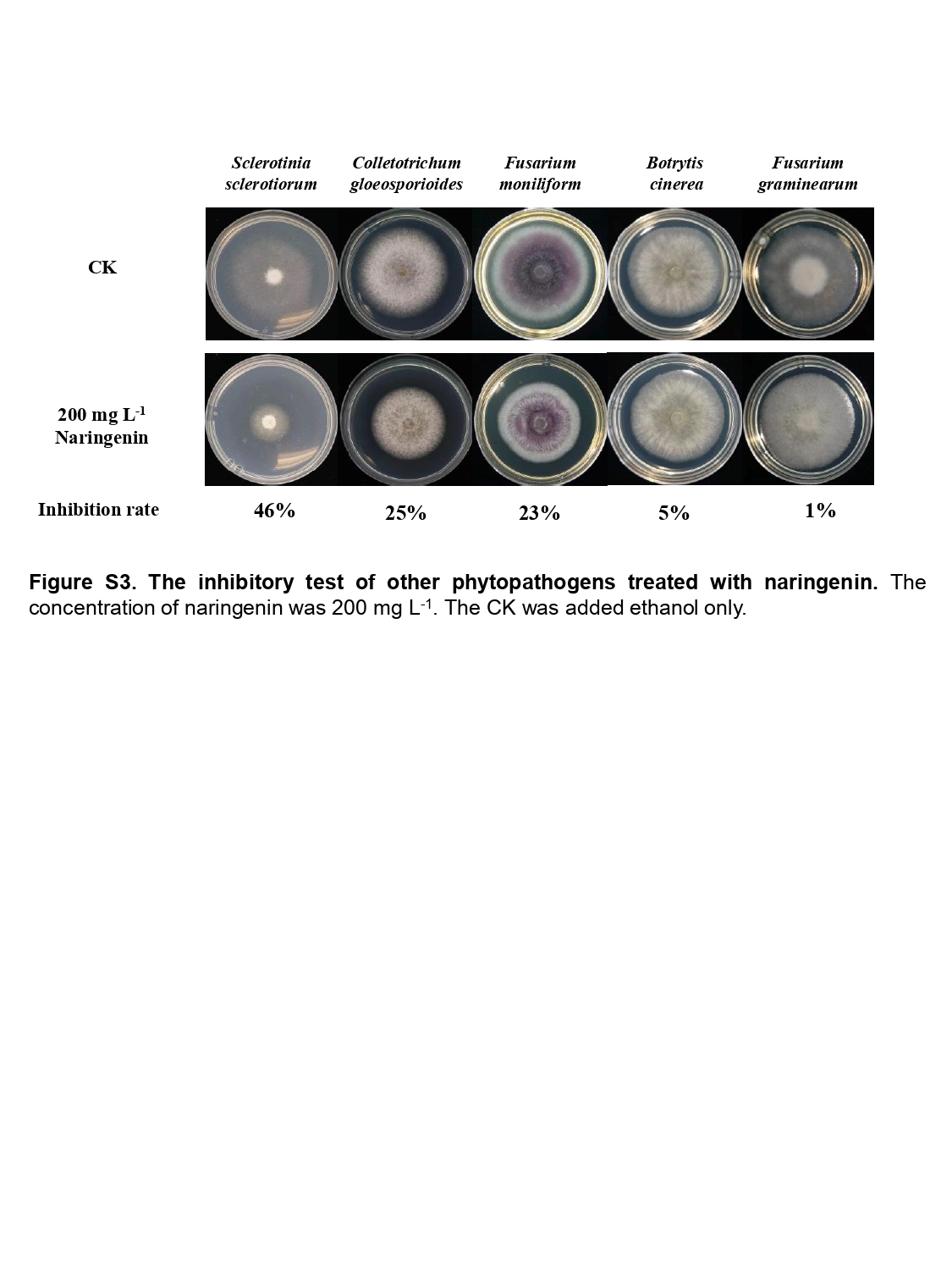

Supplement: Supplementary file 3 — Figure S3 The inhibitory test of other phytopathogens treated with naringenin. The concentration of naringenin was 200 mg/L. The control (CK) was added ethanol only. [file MPP-23-1737-s010.jpg]

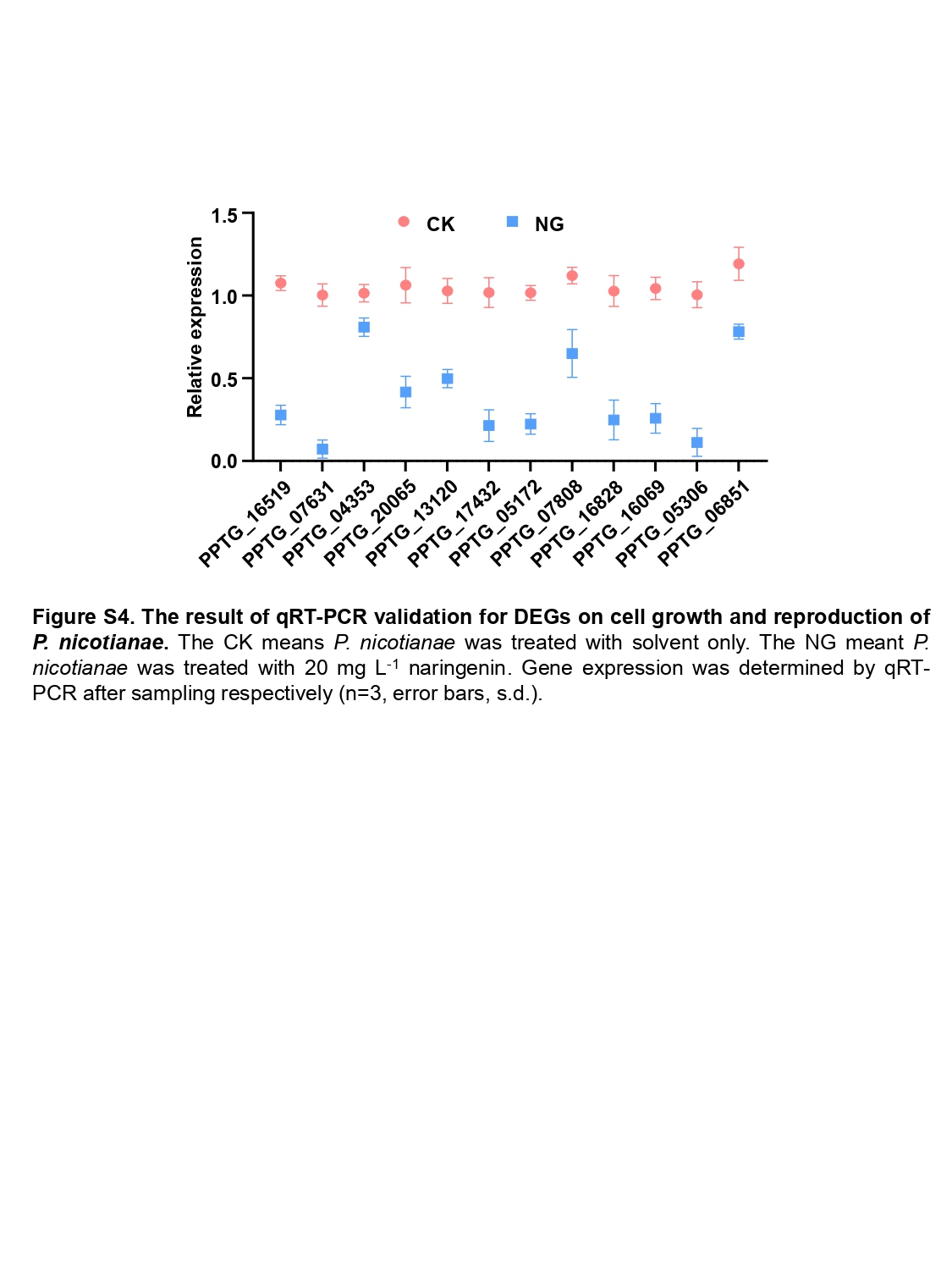

Supplement: Supplementary file 4 — Figure S4 The result of reverse transcription‐quantitative PCR (RT‐qPCR) validation for differentially expressed genes on cell growth and reproduction of Phytophthora nicotianae. The control (CK) means P. nicotianae was treated with solvent only. The NG means P. nicotianae was treated with 20 mg/L naringenin. Gene expression was determined by RT‐qPCR after sampling (n = 3, error bars, SD). [file MPP-23-1737-s007.jpg]

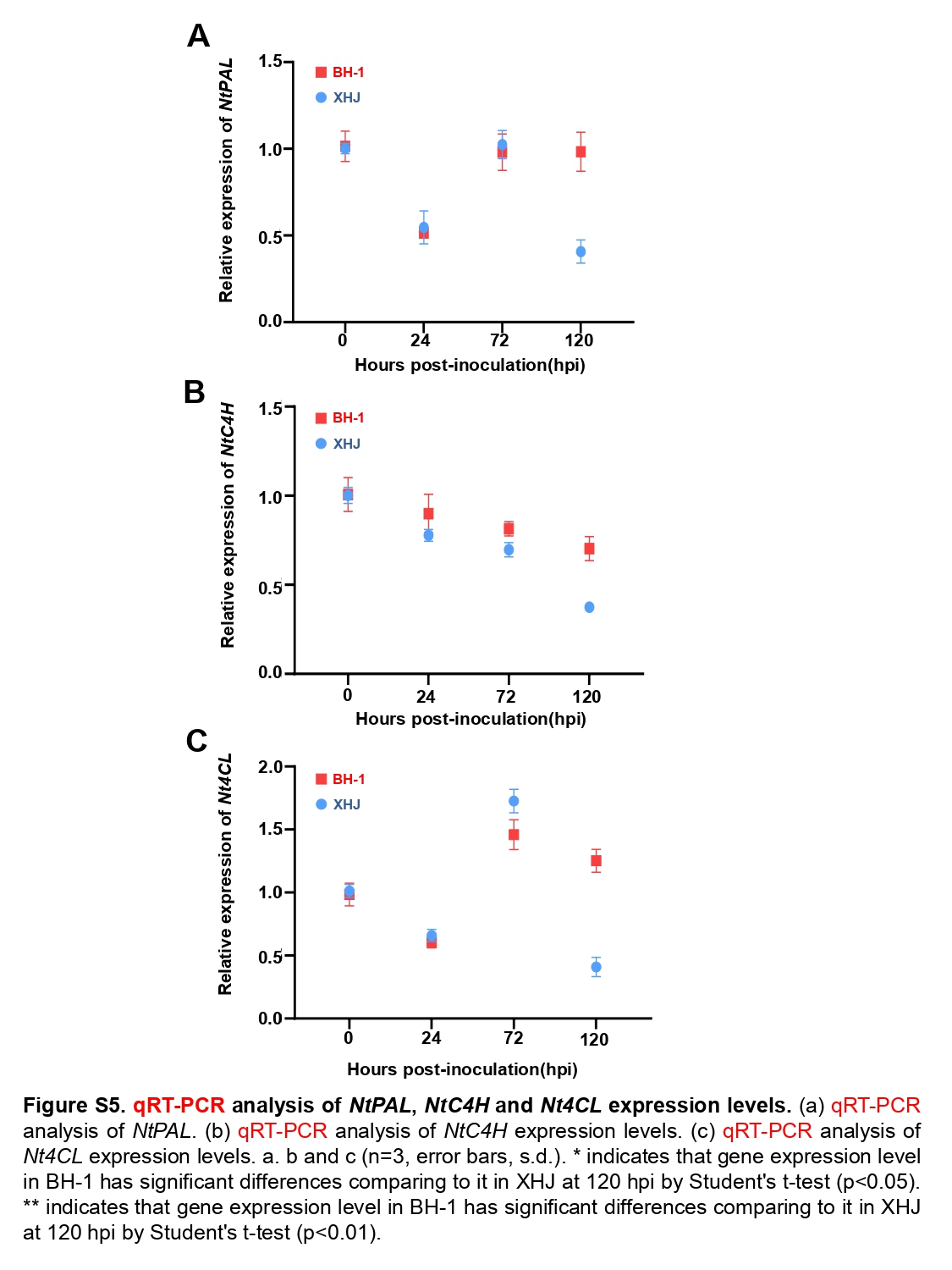

Supplement: Supplementary file 5 — Figure S5Reverse transcription‐quantitative PCR (RT‐qPCR) analysis of NtPAL, NtC4H and Nt4CL expression levels. (a) RT‐qPCR analysis of NtPAL. (b) RT‐qPCR analysis of NtC4H expression levels. (c) RT‐qPCR analysis of Nt4CL expression levels (n = 3, error bars, SD). * indicates that gene expression level in BH‐1 has differences comparing to it in XHJ at 120 postinoculation (hpi) by Student’s t test (p < 0.05). ** indicates that gene expression level in BH‐1 has significant differences comparing to it in XHJ at 120 hpi by Student’s t test (p < 0.01). [file MPP-23-1737-s014.jpg]

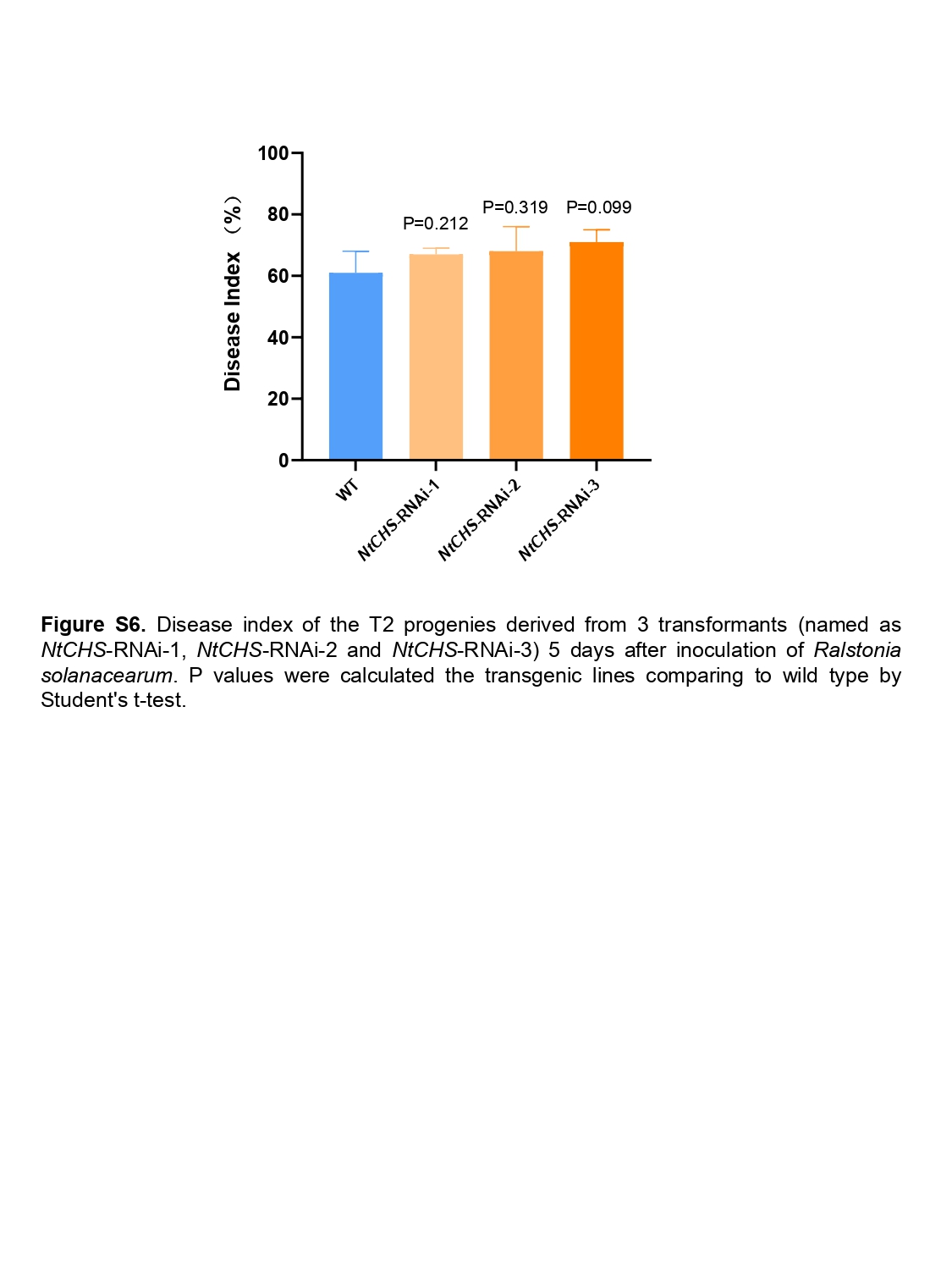

Supplement: Supplementary file 6 — Figure S6 Disease index of the T2 progenies derived from three transformants (NtCHS‐RNAi‐1, NtCHS‐RNAi‐2 and NtCHS‐RNAi‐3) 5 days after inoculation of Ralstonia solanacearum. Disease index of the transgenic lines comparing to that of the wild type was showed as p values by Student’s t test. [file MPP-23-1737-s015.jpg]

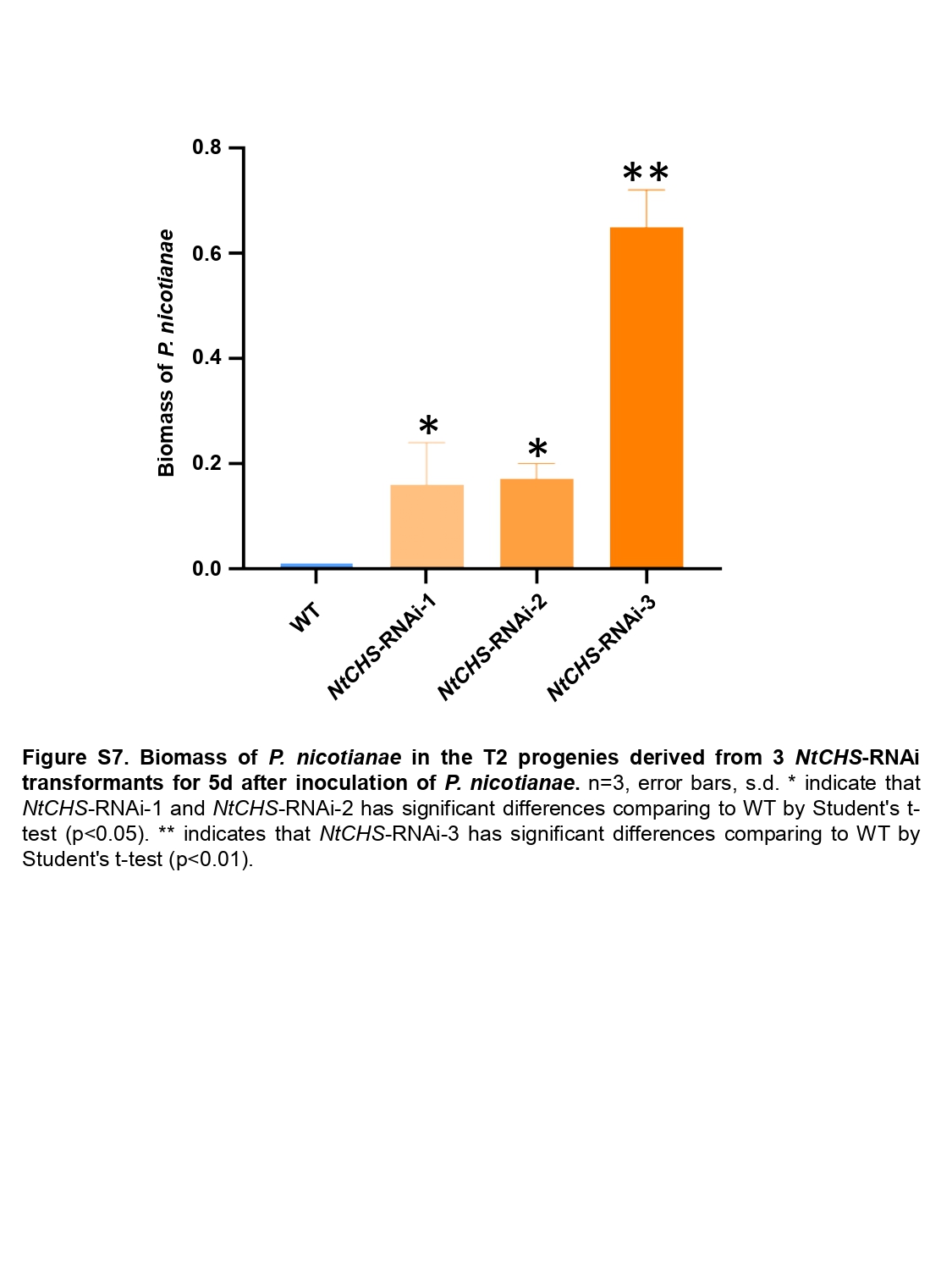

Supplement: Supplementary file 7 — Figure S7 Biomass of Phytophthora nicotianae in the T2 progenies derived from three NtCHS‐RNAi transformants for 5 days after inoculation of P. nicotianae. n = 3, error bars, SD. * indicate that NtCHS‐RNAi‐1 and NtCHS‐RNAi‐2 has differences comparing to the wild type (WT) by Student’s t test (p < 0.05). ** indicates that NtCHS‐RNAi‐3 has significant differences comparing to WT by Student’s t test (p < 0.01). [file MPP-23-1737-s005.jpg]

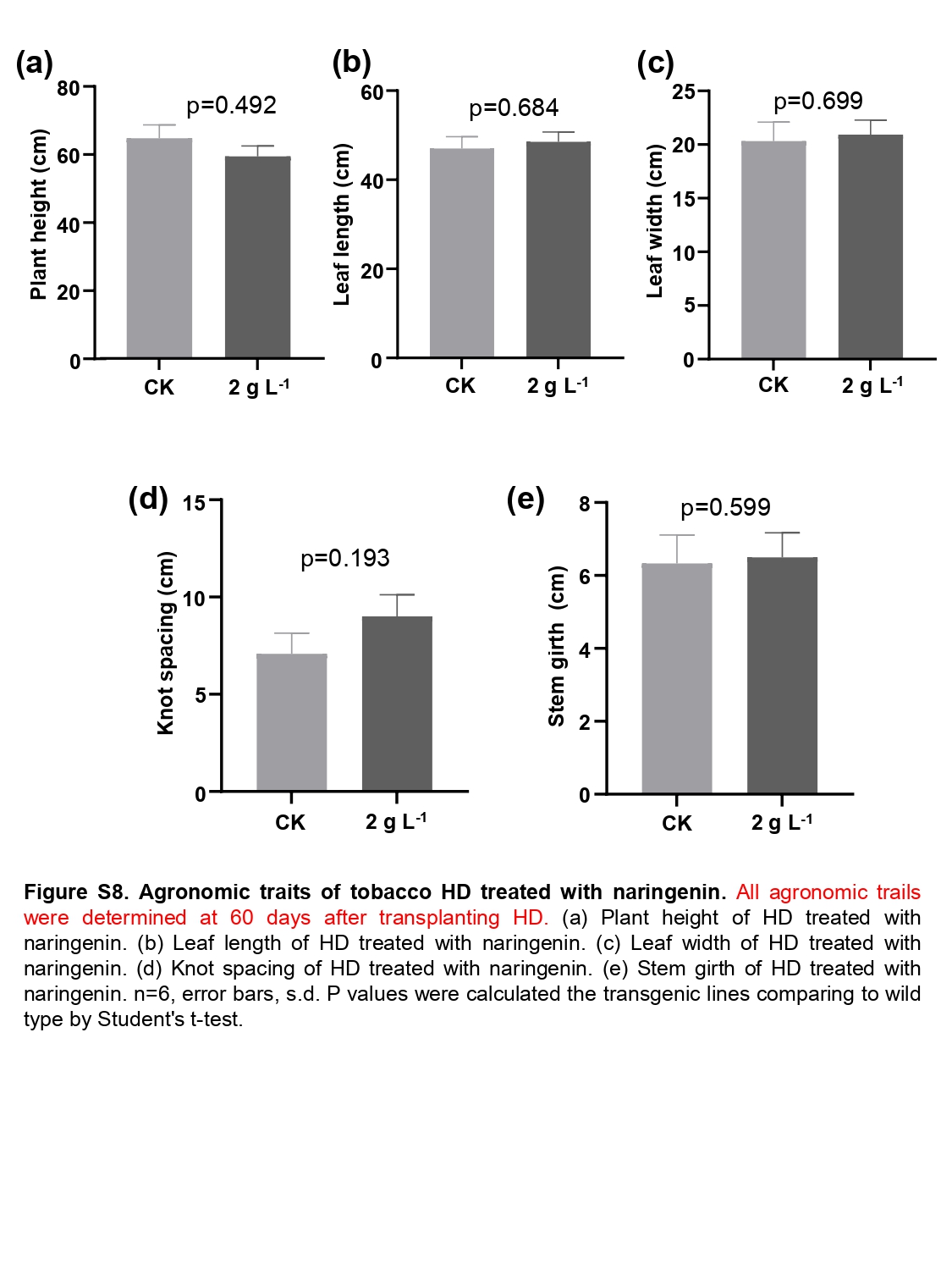

Supplement: Supplementary file 8 — Figure S8 Agronomic traits of tobacco var. Honghuadajinyuan (HD) treated with naringenin. All agronomic trails were determined at 60 days after transplanting HD. (a) Plant height of HD treated with naringenin. (b) Leaf length of HD treated with naringenin. (c) Leaf width of HD treated with naringenin. (d) Knot spacing of HD treated with naringenin. (e) Stem girth of HD treated with naringenin. n = 6, error bars, SD. p values were calculated the transgenic lines comparing to the wild type by Student’s t test. [file MPP-23-1737-s009.jpg]

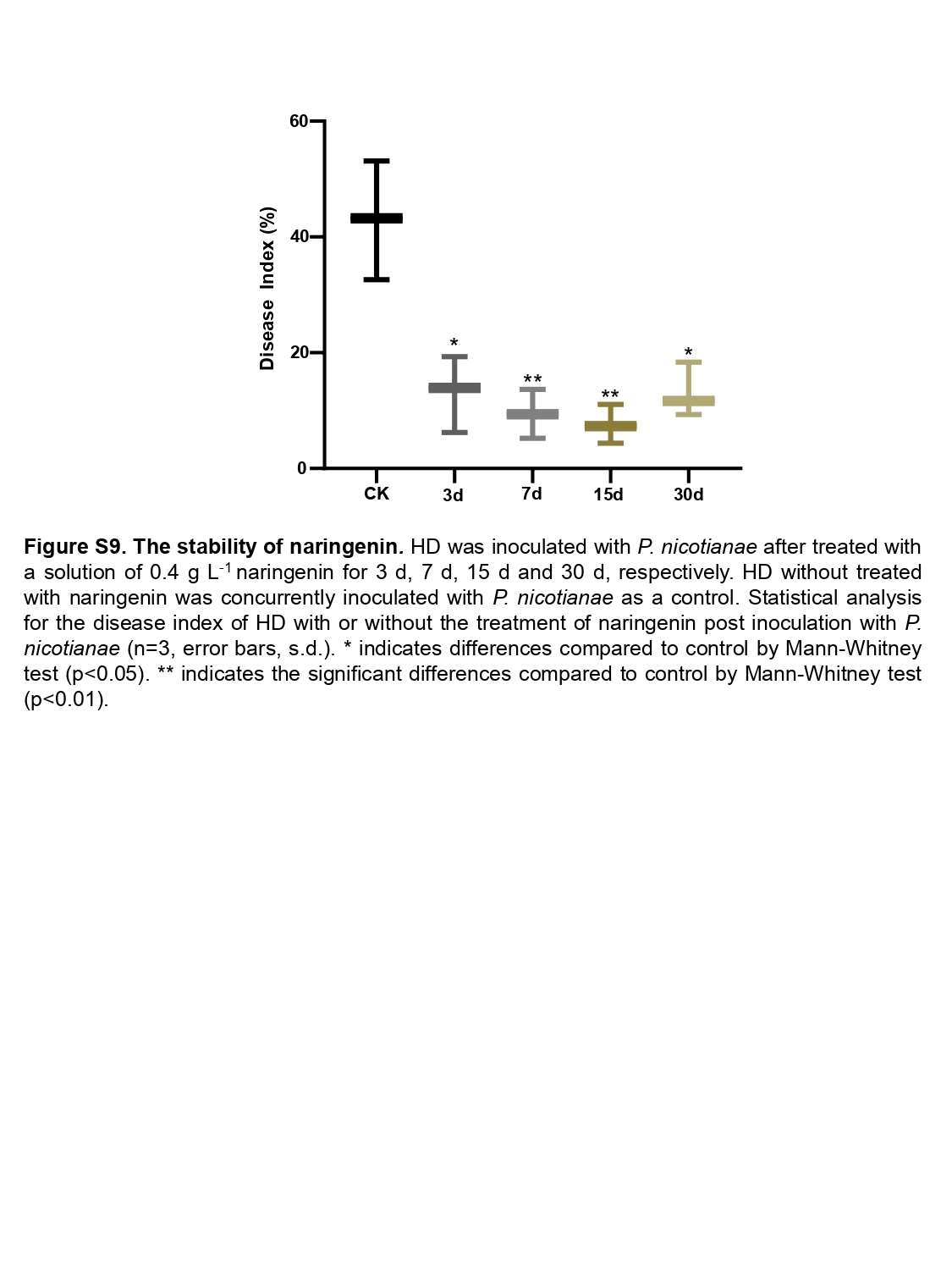

Supplement: Supplementary file 9 — Figure S9 The stability of naringenin. Honghuadajinyuan (HD) was inoculated with Phytophthora nicotianae after treated with a solution of 0.4 g/L naringenin for 3, 7, 15 and 30 days. HD not treated with naringenin was concurrently inoculated with P. nicotianae as a control. Statistical analysis for the disease index of HD with or without naringenin treatment postinoculation with P. nicotianae (n = 3, error bars, SD). * indicates that the desease index of HD treated with naringenin has differences compared to control by Mann–Whitney test (p < 0.05). ** indicates that the desease index of HD treated with naringenin has ignificant differences compared to control by Mann–Whitney test (p < 0.01). [file MPP-23-1737-s001.jpg]

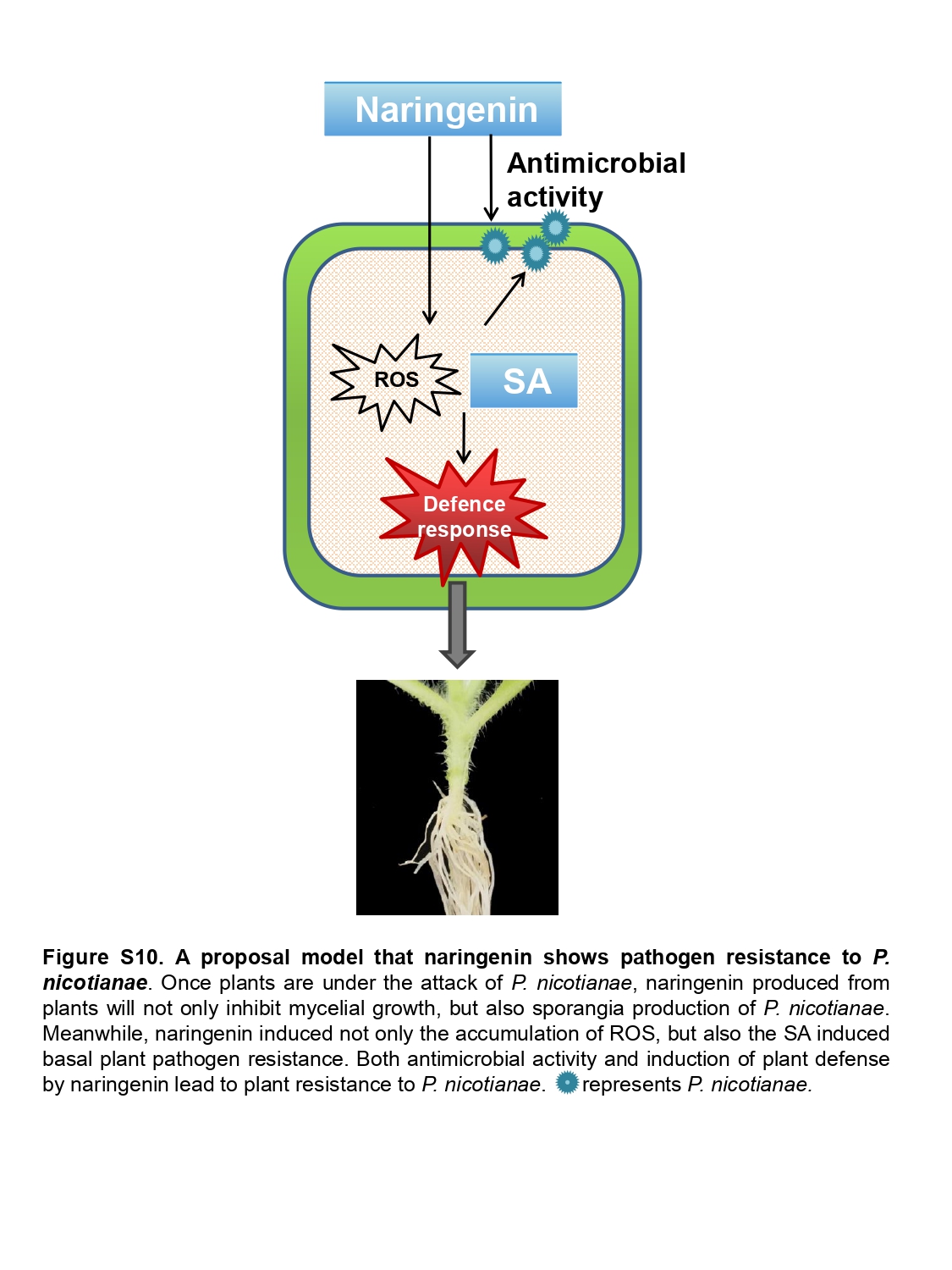

Supplement: Supplementary file 10 — Figure S10 A model of how naringenin affects pathogen resistance to Phytophthora nicotianae. Once plants are under the attack from P. nicotianae, naringenin produced from plants will not only inhibit mycelial growth, but also sporangia production of P. nicotianae. Meanwhile, naringenin induced not only the accumulation of reactive oxygen species (ROS), but also salicylic acid (SA) induced basal plant pathogen resistance. Both antimicrobial activity and induction of plant defence by naringenin lead to plant resistance to P. nicotianae. [file MPP-23-1737-s006.jpg]
