# Supplementary material for: Naringenin confers defence against Phytophthora nicotianae through antimicrobial activity and induction of pathogen resistance in tobacco
Source: Mol Plant Pathol. 2022 Sep 12;23(12):1737–50. doi: 10.1111/mpp.13255 (PMC9644278; doi:10.1111/mpp.13255)
Supplement: Supplementary file 11 — Table S1 A total of 166 metabolites was characterized by their distinct retention times and mass‐to‐charge ratios (m/z) [file MPP-23-1737-s002.docx]

**Table S1 A total of 166 metabolites was characterized by its distinct retention time and mass to charge ratio (m/z)**

| **Index** | **Compounds** | **X1** | **X2** | **X3** | **B1** | **B2** | **B3** | **VIP** | **Fold_**  **Change** |
| --- | --- | --- | --- | --- | --- | --- | --- | --- | --- |
| mws0183 | 3,4-Dihydroxybenzoic acid (Protocatechuic acid) | 1138300 | 895800 | 1150800 | 896270 | 1417500 | 1167600 | 0.20 | 1.09 |
| mws0902 | Liquiritigenin | 364 | 108 | 402 | 389 | 399 | 198 | 0.34 | 1.13 |
| mws1662 | Tectochrysin | 4371 | 9190 | 7138 | 5114 | 6764 | 10748 | 0.15 | 1.09 |
| mws0914 | Pinobanksin | 2502 | 3160 | 2128 | 2526 | 4327 | 3189 | 0.54 | 1.29 |
| pme0376 | Naringenin (5,7,4'-Trihydroxyflavanone) | 159 | 283 | 232 | 291 | 352 | 505 | 0.95 | 1.70 |
| mws0918 | Prunetin (5,4'-Dihydroxy-7-methoxyisoflavone)* | 5908 | 3808 | 3901 | 4030 | 6517 | 3799 | 0.10 | 1.05 |
| mws4160 | Wogonin (5,7-Dihydroxy-8-Methoxyflavone) | 5485 | 2706 | 2476 | 5193 | 5880 | 6033 | 0.95 | 1.60 |
| Lmmp004504 | 2'-Hydroxygenistein* | 9503 | 18122 | 11411 | 16288 | 12294 | 9778 | 0.00 | 0.98 |
| pmp000964 | Aureusidin* | 7981 | 14016 | 9586 | 15655 | 15292 | 8075 | 0.39 | 1.24 |
| mws1068 | Kaempferol (3,5,7,4'-Tetrahydroxyflavone) | 3843 | 3667 | 3701 | 3915 | 5203 | 3771 | 0.39 | 1.15 |
| mws1034 | Isosakuranetin (5,7-Dihydroxy-4'-methoxyflavanone) | 7624 | 5054 | 4424 | 12632 | 11429 | 13183 | 1.31 | 2.18 |
| pmc1990 | 4'-Hydroxy-5,7-dimethoxyflavanone | 3240100 | 3260000 | 3539400 | 3241700 | 3122300 | 3389300 | 0.16 | 0.97 |
| pme3514 | Morin | 16033 | 11211 | 13941 | 7517 | 13237 | 11262 | 0.59 | 0.78 |
| mws0463 | Hesperetin | 2193 | 6210 | 2812 | 18126 | 16627 | 7832 | 1.64 | 3.80 |
| Lmmp003109 | 3'-O-Methyl-(-)-epicatechin | 402650 | 376620 | 373060 | 200160 | 277410 | 249130 | 1.00 | 0.63 |
| pmp000106 | 5,7,4'-Trimethoxyflavone | 11042 | 38410 | 9670 | 97685 | 52613 | 20510 | 1.27 | 2.89 |
| HJAP057 | Dihydroxy-dimethoxyflavone | 3271 | 26290 | 16997 | 6553 | 3443 | 5252 | 1.02 | 0.33 |
| mws1174 | 3-O-Acetylpinobanksin | 17566 | 18060 | 14031 | 17027 | 14630 | 20298 | 0.13 | 1.05 |
| pmp000003 | Nepetin (5,7,3',4'-Tetrahydroxy-6-methoxyflavone) | 2500 | 4254 | 8242 | 5065 | 7955 | 11164 | 0.81 | 1.61 |
| Lmmn004912 | Quercetin-3-O-methyl ether | 34485 | 32342 | 37216 | 22915 | 37093 | 33645 | 0.33 | 0.90 |
| mws0066 | Isorhamnetin* | 88026 | 76429 | 93192 | 60948 | 82710 | 73794 | 0.51 | 0.84 |
| mws2627 | Tamarixetin (3,3',5,7-Tetrahydroxy-4'-Methoxyflavone)* | 17654 | 21808 | 16114 | 17740 | 20274 | 14158 | 0.19 | 0.94 |
| mws0988 | Rhamnetin (7-O-Methxyl Quercetin) | 1048 | 1869 | 1614 | 2141 | 1970 | 1636 | 0.57 | 1.27 |
| Lmfp001509 | 1-O-Galloyl-rhamnose | 132520 | 142480 | 135110 | 154690 | 123390 | 141960 | 0.08 | 1.02 |
| Cmsp006026 | Dihydrorhamnetin | 4374 | 4457 | 5690 | 3387 | 3833 | 4383 | 0.60 | 0.80 |
| mws0744 | Dihydromyricetin (Ampelopsin) | 15533 | 7368 | 20529 | 8813 | 10091 | 9746 | 0.60 | 0.66 |
| Zmgn006415 | Glabrene | 17929 | 20088 | 18337 | 22668 | 21280 | 21312 | 0.54 | 1.16 |
| mws4074 | Glabranine | 7470 | 9486 | 7196 | 7820 | 7642 | 8995 | 0.06 | 1.01 |
| pmp000107 | Monohydroxy-trimethoxyflavone | 3653 | 31391 | 3309 | 52433 | 20066 | 9279 | 1.14 | 2.13 |
| Lmsp011263 | 6-Hydroxy-5,7,4'-trimethoxyflavone | 5374 | 22583 | 27513 | 28449 | 10941 | 7497 | 0.16 | 0.85 |
| Lmjp007790 | 5-Hydroxy-3,7,4'-trimethoxyflavone | 14155 | 11874 | 10959 | 28626 | 24536 | 15481 | 1.06 | 1.86 |
| Lmgn003755 | 5,7-Dihydroxy-2'-methoxy-3',4'-methyleneoxydihydroisoflavone | 3391 | 2531 | 3042 | 1787 | 1979 | 2027 | 0.95 | 0.65 |
| Lmzp004885 | Tricin (5,7,4'-Trihydroxy-3',5'-dimethoxyflavone)* | 13831 | 13814 | 4226 | 1006 | 1166 | 450 | 2.26 | 0.08 |
| pmp000004 | 4',5,7-Trihydroxy-3',6-dimethoxyflavone (Jaceosidin)* | 3354 | 6882 | 12203 | 4997 | 8755 | 15130 | 0.41 | 1.29 |
| mws0917 | 3,7-Di-O-methylquercetin | 9 | 1339 | 3192 | 1497 | 3450 | 6330 | 1.59 | 2.48 |
| mws1003 | Laricitrin* | 102940 | 86073 | 111530 | 90716 | 156550 | 108520 | 0.35 | 1.18 |
| Lmpn007255 | Patuletin (Quercetagetin-6-methyl ether)* | 27468 | 18997 | 22349 | 20154 | 31518 | 27802 | 0.34 | 1.15 |
| Lmjn004351 | Quercetagetin-4'-Methyl Ether* | 20458 | 16663 | 19578 | 16563 | 25089 | 19349 | 0.17 | 1.08 |
| Lmfn000604 | 6-O-Galloyl-glucose* | 327580 | 285540 | 372760 | 408860 | 372870 | 442140 | 0.62 | 1.24 |
| pmb2928 | Gallic acid-4-O-glucoside* | 347390 | 417530 | 541460 | 349670 | 271050 | 294950 | 0.77 | 0.70 |
| Lmsp009872 | 5,7,3',4'-Tetramethoxyflavone | 90533 | 749160 | 168540 | 221030 | 113670 | 97623 | 0.65 | 0.43 |
| Lmcp007240 | 5,6,7,4'-Tetramethoxyflavone* | 245390 | 814490 | 242840 | 1085900 | 606290 | 413040 | 0.82 | 1.62 |
| pmp000109 | 5,7,8,4'-Tetramethoxyflavone* | 72136 | 248510 | 70259 | 338920 | 185460 | 119810 | 0.83 | 1.65 |
| Zmhp004065 | 7,8-Dihydroxy-5,6,4'-trimethoxyflavone* | 6640 | 31834 | 21684 | 61358 | 21727 | 33639 | 0.98 | 1.94 |
| mws1474 | 5,7-Dihydroxy-3',4',5'-trimethoxyflavone | 5900 | 34414 | 24302 | 24153 | 12292 | 11856 | 0.16 | 0.75 |
| pmp000006 | Eupatilin (5,7-Dihydroxy-3',4',6-Trimethoxyflavone)* | 6354 | 29654 | 19312 | 59104 | 22692 | 33259 | 1.08 | 2.08 |
| pmp000786 | Eupatorin (5,3'-Dihydroxy-6,7,4'-trimethoxyflavone)* | 6612 | 26903 | 19308 | 61689 | 18970 | 35107 | 1.07 | 2.19 |
| mws1002 | Syringetin | 1313300 | 1165500 | 1388200 | 1338700 | 1813900 | 1678200 | 0.59 | 1.25 |
| Lmgn002933 | 5,7-Dihydroxy-2',3',4'-trimethoxydihydroisoflavone | 8096 | 9022 | 8888 | 8881 | 6957 | 5456 | 0.54 | 0.82 |
| Hmhp007382 | Altisin* | 12236 | 51401 | 40698 | 105330 | 76658 | 23926 | 0.85 | 1.97 |
| pmp000008 | 5-Hydroxy-6,7,3',4'-tetramethoxyflavone | 23247 | 96677 | 120680 | 127420 | 60824 | 64645 | 0.28 | 1.05 |
| pmp000169 | Andrographidine D aglycone | 31238 | 131030 | 117210 | 256050 | 182720 | 65916 | 0.79 | 1.81 |
| Cmsn000894 | 7-O-Galloyl-D-sedoheptulose | 16444 | 19220 | 16508 | 15081 | 16859 | 14922 | 0.39 | 0.90 |
| mws0055 | Tangeretin* | 6969400 | 16119000 | 9120500 | 26966000 | 15563000 | 9420800 | 0.72 | 1.61 |
| pmp001076 | Isosinensetin* | 98070 | 907850 | 100610 | 728520 | 341570 | 201550 | 0.66 | 1.15 |
| mws1313 | Sinensetin (5,6,7,3',4'-pentamethoxyflavone)* | 3855 | 55713 | 7918 | 17805 | 6911 | 6926 | 0.26 | 0.47 |
| Lmsp010424 | 3,4',5,6,7-Pentamethoxyflavone* | 5067 | 13741 | 5594 | 23362 | 12519 | 7514 | 0.84 | 1.78 |
| Zmhp004269 | Skullcapflavone II | 7191 | 32913 | 42546 | 74110 | 44809 | 40114 | 1.07 | 1.92 |
| pmp000113 | 5-Hydroxy-6,7,8,3',4'-pentamethoxyflavone* | 79309 | 367300 | 298170 | 691380 | 410480 | 310040 | 1.01 | 1.90 |
| pmp001161 | 5-Hydroxyauranetin* | 32179 | 154660 | 135170 | 293450 | 185190 | 133440 | 1.02 | 1.90 |
| Lmjp006769 | Artemetin (5-Hydroxy-3,3',4',6,7-pentamethoxyflavone) | 2617 | 8209 | 10151 | 17017 | 23011 | 5140 | 0.92 | 2.15 |
| pmb0681 | Apigenin-8-C-Arabinoside | 28073 | 18292 | 38197 | 18630 | 14916 | 11966 | 1.01 | 0.54 |
| mws0043 | Nobiletin (5,6,7,8,3',4'-Hexamethoxyflavone) | 4244300 | 15995000 | 5090700 | 20638000 | 11579000 | 7130100 | 0.75 | 1.55 |
| pmp000114 | 5,6,7,8,3',4'-Hexamethoxyflavanone | 3709 | 10621 | 7525 | 32536 | 41672 | 6907 | 1.32 | 3.71 |
| Lmyp005841 | Chrysin-7-O-glucoside | 36037 | 43341 | 43995 | 33482 | 37780 | 32507 | 0.54 | 0.84 |
| pmp000417 | Daidzein-4'-O-glucoside | 97111 | 79456 | 73418 | 72729 | 85372 | 64088 | 0.34 | 0.89 |
| pme1587 | Daidzein-7-O-glucoside(Daidzin) | 75777 | 117890 | 84925 | 74952 | 69630 | 86095 | 0.45 | 0.83 |
| Lmyp005932 | Apigenin-6-C-fucoside | 6959 | 7096 | 5646 | 5077 | 4944 | 4683 | 0.76 | 0.75 |
| pmb3006 | Apigenin-7-O-glucoside(Cosmosiin) | 19928 | 12732 | 12328 | 8192 | 13355 | 7274 | 0.84 | 0.64 |
| pmp000117 | 3,5,6,7,8,3',4'-Heptamethoxyflavone | 96894 | 834230 | 153530 | 211560 | 214510 | 93169 | 0.45 | 0.48 |
| pme3392 | Pelargonidin-3-O-glucoside | 41063 | 50990 | 52459 | 27434 | 30610 | 32943 | 0.99 | 0.63 |
| HJN087 | Naringenin-4'-O-glucoside* | 40149 | 37281 | 41501 | 40758 | 38445 | 45545 | 0.20 | 1.05 |
| mws1179 | Naringenin-7-O-glucoside (Prunin) | 21331 | 18384 | 20507 | 21429 | 22205 | 27635 | 0.50 | 1.18 |
| Lmlp006175 | Isosalipurposide (Phlorizin Chalcone) | 33092 | 23227 | 31588 | 36144 | 23744 | 28858 | 0.02 | 1.01 |
| HJN090 | Butin-7-O-glucoside* | 57059 | 32547 | 46267 | 38782 | 45562 | 46326 | 0.04 | 0.96 |
| pmp000550 | Calycosin-7-O-glucoside | 86520 | 105060 | 88044 | 92614 | 78357 | 82362 | 0.34 | 0.91 |
| mws1172 | Trifolirhizin (Maackiain-3-O-glucoside) | 15201 | 14562 | 15289 | 7006 | 6729 | 6550 | 1.35 | 0.45 |
| pme2459 | Luteolin-7-O-glucoside (Cynaroside)* | 93874 | 94505 | 69084 | 65028 | 64833 | 79790 | 0.53 | 0.81 |
| Xmyp005654 | Kaempferol-4'-O-glucoside* | 96486 | 63484 | 78961 | 96662 | 71134 | 73509 | 0.05 | 1.01 |
| mws0045 | Quercetin-3-O-rhamnoside(Quercitrin)* | 28628 | 51874 | 4073 | 73188 | 34108 | 36263 | 1.02 | 1.70 |
| mws1361 | Astilbin | 10927 | 8021 | 9434 | 4591 | 4757 | 6329 | 1.11 | 0.55 |
| HJN041 | Epicatechin glucoside | 19380 | 16856 | 16743 | 22266 | 21474 | 23098 | 0.70 | 1.26 |
| pmn001702 | Tetahydroxyflavone-7-O-glucuronide* | 33548 | 37266 | 39184 | 17050 | 26329 | 29617 | 0.89 | 0.66 |
| Lmzn001894 | Kaempferol-3-O-glucuronide* | 61989 | 76505 | 65260 | 46036 | 46759 | 46828 | 0.90 | 0.69 |
| pmb2999 | Chrysoeriol-5-O-glucoside | 10433 | 9950 | 13886 | 9 | 9 | 9 | 4.03 | 0.00 |
| mws0091 | Quercetin-3-O-glucoside (Isoquercitrin)* | 9299 | 9520 | 10001 | 5631 | 5174 | 11428 | 0.64 | 0.77 |
| pmp001309 | 6-Hydroxykaempferol-7-O-glucoside* | 98426 | 75607 | 106180 | 105800 | 40077 | 62963 | 0.64 | 0.75 |
| Hmcn001884 | 6-Hydroxyluteolin 5-glucoside* | 18031 | 21690 | 9876 | 12314 | 9314 | 15201 | 0.50 | 0.74 |
| Lmdp003286 | Isohyperoside* | 119290 | 53110 | 78288 | 50081 | 82248 | 87437 | 0.21 | 0.88 |
| mws0061 | Quercetin-3-O-galactoside (Hyperin)* | 8765 | 7286 | 16452 | 10155 | 6793 | 12487 | 0.13 | 0.91 |
| mws0856 | Spiraeoside | 55346 | 58840 | 67213 | 28292 | 18158 | 15294 | 1.54 | 0.34 |
| pmb3019 | Chrysoeriol-7-O-homovanillic acid | 8634 | 8519 | 7993 | 8702 | 8758 | 9565 | 0.33 | 1.07 |
| Lmzp002365 | Hesperetin-7-O-glucoside | 213430 | 779690 | 78958 | 403570 | 582640 | 204270 | 0.53 | 1.11 |
| pme1398 | Delphinidin-3-O-glucoside (Mirtillin) | 20136 | 24806 | 30480 | 60458 | 26651 | 31887 | 0.74 | 1.58 |
| Xmsn002700 | Taxifolin-3'-O-glucoside | 390750 | 357740 | 349720 | 226960 | 233070 | 233440 | 1.01 | 0.63 |
| HJAP154 | Galloylisorhamnetin* | 4191 | 2111 | 7998 | 258750 | 149490 | 187780 | 2.93 | 41.68 |
| Hmmp002121 | Isorhamnetin-3-O-gallate* | 3349 | 9 | 3446 | 181200 | 159830 | 203520 | 3.36 | 80.03 |
| Lmjp003044 | Isorhamnetin-3-O-Glucoside* | 159940 | 80742 | 122880 | 113880 | 103690 | 92965 | 0.27 | 0.85 |
| Hmcp002207 | Isorhamnetin-7-O-glucoside (Brassicin)* | 43912 | 38061 | 26105 | 14319 | 12415 | 26172 | 1.17 | 0.49 |
| mws1454 | Persicoside | 115080 | 68302 | 103080 | 69148 | 87968 | 61719 | 0.57 | 0.76 |
| pme3391 | Petunidin-3-O-glucoside | 34779 | 44363 | 57291 | 60560 | 48198 | 55950 | 0.50 | 1.21 |
| HJN104 | Dihydromyricetin-3-O-glucoside | 59618 | 66009 | 63855 | 62544 | 52392 | 51178 | 0.46 | 0.88 |
| pmb3042 | Tricin-5-O-Glucoside | 32157 | 47091 | 44014 | 56220 | 47995 | 45036 | 0.52 | 1.21 |
| pme0444 | Malvidin-3-O-glucoside (Oenin) | 35612 | 28532 | 26958 | 25303 | 29360 | 33325 | 0.10 | 0.97 |
| Lmfn001584 | Methyl 4,6-di-O-galloyl-D-glucoside | 3743 | 4147 | 4909 | 5824 | 3957 | 6310 | 0.52 | 1.26 |
| pmn001642 | Kaempferol-3-O-(2''-O-acetyl)glucuronide | 56262 | 57140 | 61760 | 67752 | 64461 | 65195 | 0.49 | 1.13 |
| pmb3000 | Chrysoeriol-7-O-(6''-acetyl)glucoside | 10495 | 9874 | 7258 | 11578 | 6928 | 9104 | 0.01 | 1.00 |
| pmp000013 | Eupatilin-7-O-glucoside | 9 | 107940 | 6659 | 9 | 6443 | 9 | 1.74 | 0.06 |
| pmp000531 | Phellamurin | 41596 | 66390 | 41881 | 81898 | 73829 | 64468 | 0.84 | 1.47 |
| pmb3041 | Tricin-7-O-saccharic acid | 373680 | 427440 | 361860 | 306150 | 341920 | 247330 | 0.69 | 0.77 |
| pmp000235 | Salcolin B | 108750 | 53434 | 33798 | 22208 | 8189 | 9162 | 1.75 | 0.20 |
| pmp000234 | Salcolin A | 94333 | 39490 | 28818 | 13328 | 5856 | 6111 | 1.91 | 0.16 |
| Lmtp002942 | Apigenin-6,8-di-C-arabinoside* | 42573 | 53098 | 28812 | 102880 | 74154 | 92946 | 1.28 | 2.17 |
| Lmtp002822 | Apigenin-6-C-arabinoside-8-C-xyloside* | 51769 | 43870 | 36613 | 72475 | 70511 | 62484 | 0.96 | 1.55 |
| pmb2979 | Hesperetin-7-O-(6''-malonyl)glucoside | 67063 | 80271 | 62033 | 48640 | 58469 | 46824 | 0.76 | 0.74 |
| mws1292 | Isoschaftoside* | 13416 | 5345 | 667 | 4665 | 3349 | 9 | 1.31 | 0.41 |
| pmp000411 | Genistein-8-C-apiosyl(1→6)glucoside* | 9353 | 33572 | 770 | 3162 | 3963 | 1603 | 0.79 | 0.20 |
| Lmpp003930 | Apigenin-7-O-(6''-p-Coumaryl)glucoside | 22026 | 82134 | 12618 | 11825 | 26090 | 8621 | 0.89 | 0.40 |
| mws1066 | Naringenin-7-O-Rutinoside(Narirutin)* | 68562 | 612320 | 53776 | 185400 | 290620 | 109100 | 0.37 | 0.80 |
| mws0046 | Naringenin-7-O-Neohesperidoside(Naringin)* | 54705 | 536510 | 34889 | 151640 | 243930 | 92571 | 0.45 | 0.78 |
| Lmyp004318 | Kaempferol-3-O-(6''-p-Coumaroyl)galactoside* | 24908 | 258590 | 26492 | 148700 | 156580 | 48149 | 0.70 | 1.14 |
| Lmyp004407 | Kaempferol-3-O-(2''-p-Coumaroyl)galactoside | 27146 | 248810 | 22230 | 154780 | 160480 | 49955 | 0.78 | 1.22 |
| Hmjn004446 | Luteolin-7-O-(6''-caffeoyl)rhamnoside* | 19859 | 241790 | 26969 | 152660 | 153200 | 49293 | 0.80 | 1.23 |
| mws1290 | Kaempferol-3-O-(6''-p-coumaroyl)glucoside (Tiliroside)* | 32970 | 268080 | 31072 | 188090 | 171880 | 54599 | 0.71 | 1.25 |
| pmp001079 | Luteolin-7-O-neohesperidoside (Lonicerin) | 34438 | 85654 | 4932 | 26078 | 23950 | 16325 | 0.12 | 0.53 |
| pme1605 | Kaempferol-3-O-robinobioside(Biorobin)* | 29175 | 120970 | 14624 | 42518 | 38141 | 29966 | 0.02 | 0.67 |
| pmp000593 | Luteolin-7-O-rutinoside* | 22066 | 96234 | 9819 | 46587 | 23122 | 18894 | 0.00 | 0.69 |
| Lmsn002815 | Kaempferol-3-O-rutinoside(Nicotiflorin)* | 9810 | 3415 | 12219 | 9 | 9 | 6413 | 2.72 | 0.25 |
| Lmnp002413 | Luteolin-6-C-rhamnoside-7-O-glucoside | 31268 | 106490 | 8318 | 43088 | 30640 | 21358 | 0.02 | 0.65 |
| Lmjp002867 | Kaempferol-3-O-neohesperidoside | 39978 | 21155 | 30255 | 13353 | 17324 | 23495 | 0.92 | 0.59 |
| mws1073 | Apigenin-6,8-di-C-glucoside | 38077 | 161110 | 7213 | 4166 | 4689 | 11066 | 1.66 | 0.10 |
| mws0791 | Poncirin (Isosakuranetin-7-O-neohesperidoside) | 22111 | 239670 | 26291 | 144690 | 202140 | 41345 | 0.78 | 1.35 |
| Lmpp003789 | Cyanidin-3-O-(6''-O-p-Coumaroyl)glucoside | 32492 | 18970 | 15743 | 9493 | 16535 | 14267 | 0.86 | 0.60 |
| mws1519 | Eriodictyol-7-O-Rutinoside (Eriocitrin) | 3497 | 12849 | 2571 | 14945 | 13303 | 7977 | 1.13 | 1.92 |
| pmb3002 | Chrysoeriol-7-O-rutinoside | 75658 | 299610 | 18538 | 89145 | 90616 | 42899 | 0.06 | 0.57 |
| Lmpp003977 | Peonidin-3-O-(6''-O-p-Coumaroyl)glucoside | 75038 | 301260 | 16414 | 116850 | 103060 | 39422 | 0.10 | 0.66 |
| Lmmp003091 | Quercetin-3-O-(4''-O-glucosyl)rhamnoside* | 95283 | 177980 | 205020 | 79052 | 59902 | 73504 | 1.22 | 0.44 |
| pmb0711 | Quercetin-7-O-rutinoside* | 78116 | 235520 | 221430 | 68448 | 46946 | 83364 | 1.26 | 0.37 |
| mws0059 | Quercetin-3-O-rutinoside (Rutin)* | 23541 | 59193 | 58444 | 19694 | 19621 | 22034 | 1.16 | 0.43 |
| pmn001583 | Quercetin-3-O-robinobioside* | 36742 | 81703 | 100910 | 25892 | 24103 | 26622 | 1.37 | 0.35 |
| Lmjp002461 | Quercetin-3-O-neohesperidoside* | 82844 | 240740 | 223970 | 70180 | 61852 | 65636 | 1.30 | 0.36 |
| mws0036 | Hesperetin-7-O-rutinoside (Hesperidin)* | 2916800 | 7705000 | 1143600 | 7368600 | 7620300 | 2288400 | 0.68 | 1.47 |
| pme0001 | Hesperetin-7-O-neohesperidoside(Neohesperidin)* | 2470700 | 9966100 | 1190300 | 6972700 | 7064800 | 2179900 | 0.53 | 1.19 |
| Lmpp003662 | Delphinidin-3-O-(6''-O-p-coumaroyl)glucoside | 87630 | 154270 | 157440 | 49721 | 48239 | 53751 | 1.41 | 0.38 |
| Lmmp006598 | Apigenin-7-O-(2''-feruloyl)glucuronide | 5504 | 5963 | 5588 | 6621 | 6652 | 6591 | 0.58 | 1.16 |
| Hmjp002999 | Isorhamnetin-3-O-rutinoside (Narcissin)* | 29218 | 11885 | 29155 | 3962 | 3163 | 13436 | 1.56 | 0.29 |
| HJAP120 | Rhamnetin-3-O-Rutinoside | 54095 | 18118 | 42500 | 5406 | 13417 | 12338 | 1.55 | 0.27 |
| Lmhp003217 | 2'-Hydoxy,5-methoxyGenistein-O-rhamnosyl-glucoside* | 43277 | 26091 | 30774 | 11778 | 12648 | 19654 | 1.29 | 0.44 |
| Lmpp003815 | Petunidin-3-O-(6''-O-p-Coumaroyl)glucoside | 32960 | 23557 | 27498 | 8656 | 15662 | 5539 | 1.49 | 0.36 |
| Lmtp003677 | Quercetin-3-O-sophoroside (Baimaside) | 8748 | 53681 | 7961 | 10096 | 6108 | 14666 | 0.60 | 0.44 |
| pmp000596 | Quercetin-3-O-(2''-O-galactosyl)glucoside | 65133 | 68128 | 57516 | 31710 | 43571 | 43578 | 1.00 | 0.62 |
| pmp001310 | 6-Hydroxykaempferol-3,6-O-Diglucoside | 6063 | 56105 | 19498 | 14038 | 8044 | 10147 | 0.74 | 0.39 |
| pmb2970 | Hesperetin-5,7-di-O-glucoside | 11219 | 10086 | 11361 | 11429 | 6523 | 10516 | 0.39 | 0.87 |
| Hmln001836 | Kaempferol-3-O-(6''-Acetyl)glucosyl-(1→3)-Galactoside | 14807 | 22217 | 7582 | 14311 | 13461 | 18459 | 0.22 | 1.04 |
| HJN039 | Myricetin-3-O-(2''-galloyl-4''-acetyl)rhamnoside | 33291 | 41132 | 38964 | 36021 | 30244 | 28749 | 0.53 | 0.84 |
| Hmcp001919 | isorhamnetin-3-O-(6''-acetylglucosyl)(1→3)-glucoside | 57699 | 47800 | 50609 | 69094 | 112930 | 63919 | 0.85 | 1.58 |
| HJN075 | Pinocembrin-7-O-(2'',6''-di-O-rhamnosyl)glucoside | 4499 | 4045 | 3823 | 4847 | 3859 | 4501 | 0.24 | 1.07 |
| Lmmp002995 | Quercetin-7-O-(2''-malonyl)glucosyl-5-O-glucoside | 8671 | 9 | 788 | 782 | 1576 | 3978 | 1.06 | 0.67 |
| Lmpp003268 | Kaempferol-3-O-rutinoside-7-O-glucoside | 761 | 767 | 1534 | 1523 | 5355 | 4573 | 1.50 | 3.74 |
| pmp001105 | Kaempferol-3-O-neohesperidoside-7-O-glucoside | 9 | 9 | 9 | 9 | 5077 | 9 | 1.45 | 188.70 |
| pmb0672 | Apigenin-6-C-glucoside-7-O-(6''-feruloyl)glucoside | 5385 | 3855 | 2305 | 10762 | 6160 | 4610 | 0.98 | 1.87 |
| Lmmp002755 | Quercetin-7-O-rutinoside-4'-O-glucoside | 12161 | 10710 | 10651 | 35054 | 19886 | 32022 | 1.41 | 2.59 |
| Zmmp001564 | Petunidin-3-O-rutinoside-5-O-glucoside | 12047 | 14122 | 17499 | 5862 | 6416 | 8803 | 1.24 | 0.48 |
| pmp000130 | Natsudaidain-3-O-(5'-glucosyl-3-hydroxy-3-methylglutarate)glucoside | 13778 | 19015 | 18286 | 8738 | 11284 | 8453 | 1.10 | 0.56 |
| Hmmn002691 | Eriodictyol-5,3'-Di-O-rutinoside | 15495 | 14831 | 6497 | 8058 | 12016 | 8087 | 0.39 | 0.76 |
| Hmmp002240 | Isorhamnetin-3-O-rutinoside-7-O-(2''-O-glucosyl)glucuronate | 6606 | 8318 | 4966 | 13325 | 14159 | 13192 | 1.25 | 2.05 |
