# Supplementary material for: Naringenin confers defence against Phytophthora nicotianae through antimicrobial activity and induction of pathogen resistance in tobacco
Source: Mol Plant Pathol. 2022 Sep 12;23(12):1737–50. doi: 10.1111/mpp.13255 (PMC9644278; doi:10.1111/mpp.13255)
Supplement: Supplementary file 12 — Table S2 Significant up‐regulated metabolites in Beinhart 1000‐1 (BH‐1) relative to Xiaohuangjin (XHJ) [file MPP-23-1737-s013.docx]

**Table S2 Significant upregulated metabolites in BH-1 relative to XHJ**

|  | **Index** | **Compounds** | **VIP** | **Fold_Change** | **Type** |
| --- | --- | --- | --- | --- | --- |
| **Others** | Lmtp002942 | Apigenin-6,8-di-C-arabinoside* | 1.28 | 2.17 | up |
| **Flavonoid** | pmp000106 | 5,7,4'-Trimethoxyflavone | 1.27 | 2.89 | up |
|  | pmp000107 | Monohydroxy-trimethoxyflavone | 1.14 | 2.13 | up |
|  | pmp000006 | Eupatilin (5,7-Dihydroxy-3',4',6-Trimethoxyflavone)* | 1.08 | 2.08 | up |
|  | pmp000786 | Eupatorin (5,3'-Dihydroxy-6,7,4'-trimethoxyflavone)* | 1.07 | 2.19 | up |
| **Flavonol** | mws0917 | 3,7-Di-O-methylquercetin | 1.59 | 2.48 | up |
|  | HJAP154 | Galloylisorhamnetin* | 2.93 | 41.68 | up |
|  | Hmmp002121 | Isorhamnetin-3-O-gallate* | 3.36 | 80.03 | up |
|  | Lmpp003268 | Kaempferol-3-O-rutinoside-7-O-glucoside | 1.50 | 3.74 | up |
|  | pmp001105 | Kaempferol-3-O-neohesperidoside-7-O-glucoside | 1.45 | 188.70 | up |
|  | Lmmp002755 | Quercetin-7-O-rutinoside-4'-O-glucoside | 1.41 | 2.59 | up |
|  | Hmmp002240 | Isorhamnetin-3-O-rutinoside-7-O-(2''-O-glucosyl)glucuronate | 1.25 | 2.05 | up |
| **Flavanone** | mws1034 | Isosakuranetin (5,7-Dihydroxy-4'-methoxyflavanone) | 1.31 | 2.18 | up |
|  | mws0463 | Hesperetin | 1.64 | 3.80 | up |
|  | pmp000114 | 5,6,7,8,3',4'-Hexamethoxyflavanone | 1.32 | 3.71 | up |
