# Supplementary material for: Naringenin confers defence against Phytophthora nicotianae through antimicrobial activity and induction of pathogen resistance in tobacco
Source: Mol Plant Pathol. 2022 Sep 12;23(12):1737–50. doi: 10.1111/mpp.13255 (PMC9644278; doi:10.1111/mpp.13255)
Supplement: Supplementary file 13 — Table S3 A total of 16 flavanones were characterized in metabolite analysis [file MPP-23-1737-s008.docx]

**Table S3 A total of 16 flavanones was characterized in metabolites analysis**

| **Index** | **Molecular Weight (Da)** | **Formula** | **Compounds** | **Fold_Change** |
| --- | --- | --- | --- | --- |
| mws1454 | 478.15 | C23H26O11 | Persicoside | 0.76 |
| Hmmn002691 | 904.29 | C39H52O24 | Eriodictyol-5,3'-Di-O-rutinoside | 0.76 |
| mws0046 | 580.18 | C27H32O14 | Naringenin-7-O-Neohesperidoside(Naringin)* | 0.78 |
| mws1066 | 580.18 | C27H32O14 | Naringenin-7-O-Rutinoside(Narirutin)* | 0.80 |
| mws4074 | 324.14 | C20H20O4 | Glabranine | 1.01 |
| mws0902 | 256.07 | C15H12O4 | Liquiritigenin | 1.13 |
| mws1179 | 434.12 | C21H22O10 | Naringenin-7-O-glucoside (Prunin) | 1.18 |
| pme0001 | 610.19 | C28H34O15 | Hesperetin-7-O-neohesperidoside(Neohesperidin)* | 1.19 |
| mws0791 | 594.20 | C28H34O14 | Poncirin (Isosakuranetin-7-O-neohesperidoside) | 1.35 |
| mws0036 | 610.19 | C28H34O15 | Hesperetin-7-O-rutinoside (Hesperidin)* | 1.47 |
| pme0376 | 272.07 | C15H12O5 | Naringenin (5,7,4'-Trihydroxyflavanone) | 1.70 |
| pmp000169 | 358.11 | C19H18O7 | Andrographidine D aglycone | 1.81 |
| mws1519 | 596.17 | C27H32O15 | Eriodictyol-7-O-Rutinoside (Eriocitrin) | 1.92 |
| mws1034 | 286.08 | C16H14O5 | Isosakuranetin (5,7-Dihydroxy-4'-methoxyflavanone) | 2.18 |
| pmp000114 | 404.15 | C21H24O8 | 5,6,7,8,3',4'-Hexamethoxyflavanone | 3.71 |
| mws0463 | 302.08 | C16H14O6 | Hesperetin | 3.80 |
