# Supplementary material for: Naringenin confers defence against Phytophthora nicotianae through antimicrobial activity and induction of pathogen resistance in tobacco
Source: Mol Plant Pathol. 2022 Sep 12;23(12):1737–50. doi: 10.1111/mpp.13255 (PMC9644278; doi:10.1111/mpp.13255)
Supplement: Supplementary file 14 — Table S4 A total of 44 flavonols were characterized in metabolite analysis [file MPP-23-1737-s016.docx]

**Table S4 A total of 44 flavonols was characterized in metabolites analysis**

| **Index** | **Molecular Weight (Da)** | **Formula** | **Compounds** | **Fold_Change** |
| --- | --- | --- | --- | --- |
| mws0856 | 464.10 | C21H20O12 | Spiraeoside | 0.34 |
| pmn001583 | 610.15 | C27H30O16 | Quercetin-3-O-robinobioside* | 0.35 |
| Lmjp002461 | 610.15 | C27H30O16 | Quercetin-3-O-neohesperidoside* | 0.36 |
| pmb0711 | 610.15 | C27H30O16 | Quercetin-7-O-rutinoside* | 0.37 |
| pmp001310 | 626.15 | C27H30O17 | 6-Hydroxykaempferol-3,6-O-Diglucoside | 0.39 |
| mws0059 | 610.15 | C27H30O16 | Quercetin-3-O-rutinoside (Rutin)* | 0.43 |
| Lmtp003677 | 626.15 | C27H30O17 | Quercetin-3-O-sophoroside (Baimaside) | 0.44 |
| Lmmp003091 | 610.15 | C27H30O16 | Quercetin-3-O-(4''-O-glucosyl)rhamnoside* | 0.44 |
| pmp000117 | 432.14 | C22H24O9 | 3,5,6,7,8,3',4'-Heptamethoxyflavone | 0.48 |
| Hmcp002207 | 478.11 | C22H22O12 | Isorhamnetin-7-O-glucoside (Brassicin)* | 0.49 |
| pmp000130 | 886.27 | C39H50O23 | Natsudaidain-3-O-(5'-glucosyl-3-hydroxy-3-methylglutarate)glucoside | 0.56 |
| Lmjp002867 | 594.16 | C27H30O15 | Kaempferol-3-O-neohesperidoside | 0.59 |
| pmp000596 | 626.15 | C27H30O17 | Quercetin-3-O-(2''-O-galactosyl)glucoside | 0.62 |
| Lmmp002995 | 712.15 | C30H32O20 | Quercetin-7-O-(2''-malonyl)glucosyl-5-O-glucoside | 0.67 |
| pme1605 | 594.16 | C27H30O15 | Kaempferol-3-O-robinobioside(Biorobin)* | 0.67 |
| Lmzn001894 | 462.08 | C21H18O12 | Kaempferol-3-O-glucuronide* | 0.69 |
| pmp001309 | 464.10 | C21H20O12 | 6-Hydroxykaempferol-7-O-glucoside* | 0.75 |
| mws0091 | 464.10 | C21H20O12 | Quercetin-3-O-glucoside (Isoquercitrin)* | 0.77 |
| pme3514 | 302.04 | C15H10O7 | Morin | 0.78 |
| Cmsp006026 | 318.07 | C16H14O7 | Dihydrorhamnetin | 0.80 |
| HJN039 | 658.12 | C30H26O17 | Myricetin-3-O-(2''-galloyl-4''-acetyl)rhamnoside | 0.84 |
| mws0066 | 316.06 | C16H12O7 | Isorhamnetin* | 0.84 |
| Lmjp003044 | 478.11 | C22H22O12 | Isorhamnetin-3-O-Glucoside* | 0.85 |
| Lmdp003286 | 464.10 | C21H20O12 | Isohyperoside* | 0.88 |
| Lmmn004912 | 316.06 | C16H12O7 | Quercetin-3-O-methyl ether | 0.90 |
| mws0061 | 464.10 | C21H20O12 | Quercetin-3-O-galactoside (Hyperin)* | 0.91 |
| Hmln001836 | 652.16 | C29H32O17 | Kaempferol-3-O-(6''-Acetyl)glucosyl-(1→3)-Galactoside | 1.04 |
| pmn001642 | 504.09 | C23H20O13 | Kaempferol-3-O-(2''-O-acetyl)glucuronide | 1.13 |
| mws1068 | 286.05 | C15H10O6 | Kaempferol (3,5,7,4'-Tetrahydroxyflavone) | 1.15 |
| Lmpn007255 | 332.05 | C16H12O8 | Patuletin (Quercetagetin-6-methyl ether)* | 1.15 |
| mws1003 | 332.05 | C16H12O8 | Laricitrin* | 1.18 |
| mws1290 | 594.14 | C30H26O13 | Kaempferol-3-O-(6''-p-coumaroyl)glucoside (Tiliroside)* | 1.25 |
| mws0988 | 316.06 | C16H12O7 | Rhamnetin (7-O-Methxyl Quercetin) | 1.27 |
| Hmcp001919 | 682.17 | C30H34O18 | isorhamnetin-3-O-(6''-acetylglucosyl)(1→3)-glucoside | 1.58 |
| mws0055 | 372.12 | C20H20O7 | Tangeretin* | 1.61 |
| mws0045 | 448.10 | C21H20O11 | Quercetin-3-O-rhamnoside(Quercitrin)* | 1.70 |
| pmp001161 | 388.12 | C20H20O8 | 5-Hydroxyauranetin* | 1.90 |
| Hmmp002240 | 962.26 | C40H50O27 | Isorhamnetin-3-O-rutinoside-7-O-(2''-O-glucosyl)glucuronate | 2.05 |
| mws0917 | 330.07 | C17H14O7 | 3,7-Di-O-methylquercetin | 2.48 |
| Lmmp002755 | 772.21 | C33H40O21 | Quercetin-7-O-rutinoside-4'-O-glucoside | 2.59 |
| Lmpp003268 | 756.21 | C33H40O20 | Kaempferol-3-O-rutinoside-7-O-glucoside | 3.74 |
| HJAP154 | 468.07 | C23H16O11 | Galloylisorhamnetin* | 41.68 |
| Hmmp002121 | 468.11 | C23H16O11 | Isorhamnetin-3-O-gallate* | 80.03 |
| pmp001105 | 756.21 | C33H40O20 | Kaempferol-3-O-neohesperidoside-7-O-glucoside | 188.70 |
