# Supplementary material for: Naringenin confers defence against Phytophthora nicotianae through antimicrobial activity and induction of pathogen resistance in tobacco
Source: Mol Plant Pathol. 2022 Sep 12;23(12):1737–50. doi: 10.1111/mpp.13255 (PMC9644278; doi:10.1111/mpp.13255)
Supplement: Supplementary file 15 — Table S5 The inhibitory activity of the different flavonoids on Phytophthora nicotianae [file MPP-23-1737-s012.docx]

**Table S5 The inhibitory activity of the different flavonoids on *P. nicotianae***

|  | **Naringenin** | **Liquiritigenin** | **Hesperetin** | **Kaempferol** | **Quercetin** | **Rutinum** |
| --- | --- | --- | --- | --- | --- | --- |
| **EC_50_（mg L^-1^）** | 22.01±0.70 | 51.43±0.67 | 30.20±0.80 | >400 | >200 | >200 |
| **EC_90_（mg L^-1^）** | 186.67±9.82 | 253.67±14.52 | 576.33±25.69 | >>400 | >>200 | >>200 |

EC_50_，concentration for 50% of maximal effect；EC_90_，concentration for 90% of maximal effect； > and >>，Greater than and far greater than.
