# Supplementary material for: Naringenin confers defence against Phytophthora nicotianae through antimicrobial activity and induction of pathogen resistance in tobacco
Source: Mol Plant Pathol. 2022 Sep 12;23(12):1737–50. doi: 10.1111/mpp.13255 (PMC9644278; doi:10.1111/mpp.13255)
Supplement: Supplementary file 16 — Table S6 The inhibitory test of the three pathogens and sporangia production of Phytophthora nicotianae treated with naringenin [file MPP-23-1737-s004.docx]

**Table S6 The inhibitory test of other three pathogens and sporangia production of *P. nicotianae* treated with naringenin**

|  | ***Phytophthora capsici*** | ***Pythium aphanidermatum*** | ***Pythium ultimum*** | **Sporangia production** |
| --- | --- | --- | --- | --- |
| **EC_50_（mg L^-1^）** | 50.11±1.89 | 96.67±2.46 | 83.04±2.48 | 2.01±0.15 |
| **EC_90_（mg L^-1^）** | 807.33±148.57 | 234.67±12.71 | 221±24.03 | 6.62±0.23 |
