# Supplementary material for: Naringenin confers defence against Phytophthora nicotianae through antimicrobial activity and induction of pathogen resistance in tobacco
Source: Mol Plant Pathol. 2022 Sep 12;23(12):1737–50. doi: 10.1111/mpp.13255 (PMC9644278; doi:10.1111/mpp.13255)
Supplement: Supplementary file 17 — Table S7 The inhibitory activity of naringenin on Ralstonia solancearum [file MPP-23-1737-s018.docx]

**Table S7 The inhibitory activity of naringenin on *Ralstonia solancearum***

| **Concentration**  **（mg L^-1^）** | **0** | **12.5** | **25** | **50** | **100** | **200** | **400** |
| --- | --- | --- | --- | --- | --- | --- | --- |
| **Inhibition rate**  **（%）** | 0.00 | 2.24 | 2.52 | 3.63 | 4.76 | 5.28 | 6.31 |
