# Supplementary material for: Naringenin confers defence against Phytophthora nicotianae through antimicrobial activity and induction of pathogen resistance in tobacco
Source: Mol Plant Pathol. 2022 Sep 12;23(12):1737–50. doi: 10.1111/mpp.13255 (PMC9644278; doi:10.1111/mpp.13255)
Supplement: Supplementary file 18 — Table S8 All primer sequences used for this research [file MPP-23-1737-s011.docx]

**Table S8 All primers sequence used for this research**

| **Name** | **Primers Sequence** |
| --- | --- |
| Actin-F | CAAGGAAATCACCGCTTTGG |
| Actin-R | AAGGGATGCGAGGATGGA |
| WS21-F | CTCCAGAACGTGTACATCCG |
| WS21-R | TAGCGCCCTTCTCCTCAG |
| NtPAL-qF | CAACTTCCAAGGCACTCCTATC |
| NtPAL-qR | CAGTGAGATTAGAGGGCAAACC |
| NtC4H-qF | GCTTGGCGGGTTTGATATTC |
| NtC4H-qR | AACGTGCTTCTCCTCTTCG |
| Nt4CL-qF | GCGCTTCATATTTAGGAGCC |
| Nt4CL-qR | CCTTCACTTTGCCCACAAG |
| NtCHS-qF | GAGTCCTTGTTGTTTGTTCAG |
| NtCHS-qR | AGTAAGTGGAATGTAAGCCC |
| NtCHS-F | AAAAAGCAGGCTTCGTATCACTAATAGCGAGCAT |
| NtCHS-R | AGAAAGCTGGGTCGGGCATGTCTACACCAC |
| RNAi-F | CGATTTGTGTACGCCCGACAGTC |
| RNAi-R | TCGTTATGTTTATCGGCACTTTG |
| PPTG_16519-F | AGGACAACAACCTGGACTCTGA |
| PPTG_16519-R | GCCAACTGCTGCTGATACG |
| PPTG_07631-F | ACGACTGCCCTGACTACTTC |
| PPTG_07631-R | GTTCTTGAGCACTTCATCCC |
| PPTG_04353-F | TTACGCTTCGGTGATAGTGC |
| PPTG_04353-R | GGAGACAAACAGTCCGATGAA |
| PPTG_20065-F | TGGCTTCCAACACCATCAT |
| PPTG_20065-R | CCAGTCCACTTGAACATCCC |
| PPTG_13120-F | CTGCCCATTAACCTCGTCG |
| PPTG_13120-R | CCTACCCAAATCGCCCTAC |
| PPTG_17432-F | AATAGCCACAACAAGTACACCT |
| PPTG_17432-R | TTCGCATAGACAACTAACCAG |
| PPTG_05172-F | CAGACGCCAAGTTCCTACC |
| PPTG_05172-R | AACGCATTCCAATCCAGTT |
| PPTG_07808-F | ACGCAGCCGTGTAGAATGG |
| PPTG_07808-R | TGGCAGTGGCAAAGTGAGC |
| PPTG_16828-F | AATGGACATGGCGATACTGA |
| PPTG_16828-R | ATGAGGTTTCGGACTGGAAT |
| PPTG_16069-F | ACTGCCGTTCGTTACTACAA |
| PPTG_16069-R | GAGCTTCACTGACAGGGTTC |
| PPTG_05306-F | GCTGCCAGATCACGGACTT |
| PPTG_05306-R | TCATACACCATTGCTGCACTAAC |
| PPTG_06851-F | CTCACGTCGTATCATAATCACC |
| PPTG_06851-R | GTCCGTCAACTTGGCATCT |
| *NtICS*1-F | TTACTATCCAGTGCCAAGGAC |
| *NtICS*1-R | TCTGTTTAGAAGCCACTTAACC |
| *NtEDS*-F | GAAATGCAGGAGAGCTTAGAG |
| *NtEDS*-R | CTCTTGTACTCAGGCCTAAGTC |
| *NtPR1*-F | ATGCCTATGTGGGTGACGAAG |
| *NtPR1*-R | TCTGTTGGCCTTAGGGTTGAG |
| *SAR8.2*-F | CAAGGGAGATGTCTAAGGCGG |
| *SAR8.2*-R | ACTTTGCCGGCGACTTTGCCTC |
